# Single-cell CRISPR screens in vivo map T cell fate regulomes in cancer

**DOI:** 10.1038/s41586-023-06733-x

**Published:** 2023-11-15

**Authors:** Peipei Zhou, Hao Shi, Hongling Huang, Xiang Sun, Sujing Yuan, Nicole M. Chapman, Jon P. Connelly, Seon Ah Lim, Jordy Saravia, Anil KC, Shondra M. Pruett-Miller, Hongbo Chi

**Affiliations:** 1 Department of Immunology, St. Jude Children’s Research Hospital, Memphis, TN USA; 2 Center for Advanced Genome Engineering, St. Jude Children’s Research Hospital, Memphis, TN USA

**Keywords:** Cytotoxic T cells, Tumour immunology

## Abstract

CD8^+^ cytotoxic T cells (CTLs) orchestrate antitumour immunity and exhibit inherent heterogeneity^1,2^, with precursor exhausted T (T_pex_) cells but not terminally exhausted T (T_ex_) cells capable of responding to existing immunotherapies^3–7^. The gene regulatory network that underlies CTL differentiation and whether T_ex_ cell responses can be functionally reinvigorated are incompletely understood. Here we systematically mapped causal gene regulatory networks using single-cell CRISPR screens in vivo and discovered checkpoints for CTL differentiation. First, the exit from quiescence of T_pex_ cells initiated successive differentiation into intermediate T_ex_ cells. This process is differentially regulated by IKAROS and ETS1, the deficiencies of which dampened and increased mTORC1-associated metabolic activities, respectively. IKAROS-deficient cells accumulated as a metabolically quiescent T_pex_ cell population with limited differentiation potential following immune checkpoint blockade (ICB). Conversely, targeting ETS1 improved antitumour immunity and ICB efficacy by boosting differentiation of T_pex_ to intermediate T_ex_ cells and metabolic rewiring. Mechanistically, TCF-1 and BATF are the targets for IKAROS and ETS1, respectively. Second, the RBPJ–IRF1 axis promoted differentiation of intermediate T_ex_ to terminal T_ex_ cells. Accordingly, targeting RBPJ enhanced functional and epigenetic reprogramming of T_ex_ cells towards the proliferative state and improved therapeutic effects and ICB efficacy. Collectively, our study reveals that promoting the exit from quiescence of T_pex_ cells and enriching the proliferative T_ex_ cell state act as key modalities for antitumour effects and provides a systemic framework to integrate cell fate regulomes and reprogrammable functional determinants for cancer immunity.

## Abstract (web summary)

Analyses of causal gene regulatory networks have identified key checkpoints mediating the progressive differentiation of CD8^+^ cytotoxic T cells, findings that have implications for anticancer immunotherapies such as adoptive cell therapy and immune checkpoint blockade.

## Main

Immunotherapies such as adoptive cell therapy and ICB represent effective approaches in treating cancer^8^. However, the poor persistence and proliferative capacity of T cells in the tumour microenvironment (TME) limit immunotherapeutic efficacy^8^. Furthermore, although T_ex_ cells are the major intratumoral CTL population and directly kill tumours, they gradually lose proliferative capacity and, unlike T_pex_ cells, are unresponsive to existing immunotherapies^5–7,9^. Thus, there is a need to systemically interrogate the regulatory circuitry that underlies T_pex_ to T_ex_ cell differentiation and identify strategies to functionally reinvigorate T_ex_ cells.

Forward genetic screens enable the discovery of key immuno-oncology targets^10^. Most screening approaches rely on cell fitness or established markers, which limits their abilities for unbiased biological discovery. By contrast, single-cell CRISPR (scCRISPR) screening methods—which combine pooled genetic perturbations with single-cell RNA sequencing (scRNA-seq)—are permissive for transcriptome profiling following individual genetic perturbations in a complex cellular pool. They also enable precise mapping of co-functional modules and gene expression programmes^10^. Large-scale in vivo scCRISPR screening has not yet been used for unbiased target discovery or network reconstruction in primary immune cells.

## scCRISPR screens of intratumoral CTL fate

**Extended Data Fig. 1:**
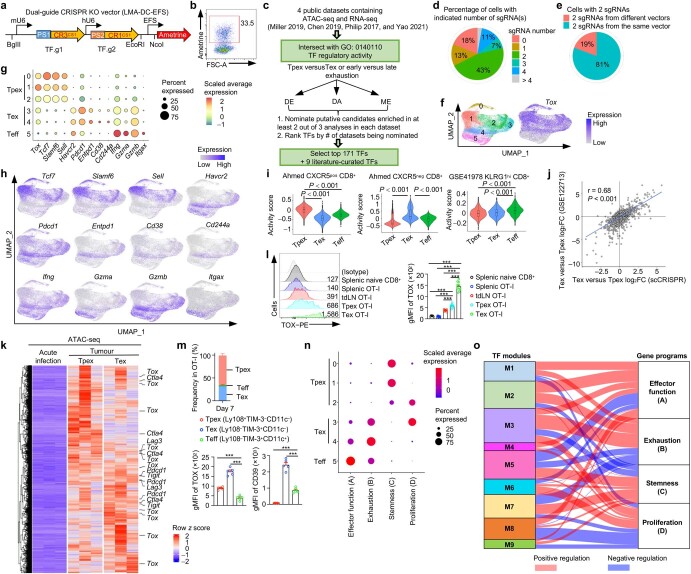
scCRISPR screening and molecular diversity of intratumoral CTLs. **a**, Schematic of the dual sgRNA CRISPR KO vector used for direct-capture Perturb-seq. PS, protospacer sequence. TF.g1, transcription factor (TF) guide RNA 1. TF.g2, TF guide RNA 2. CR, constant region. CS, capture sequence. EFS, EF1α short promoter. **b**, Percentage of dual sgRNA CRISPR KO vector-transduced OT-I cells (Ametrine^+^). **c**, Bioinformatic approach that nominated the 180 TF targets in the direct capture Perturb-seq library (see Methods). DE, differential expression. DA, differentially accessibility of chromatin state; ME, motif enrichment. **d**,**e**, Percentage of intratumoral OT-I cells with the indicated number of sgRNA(s) detected (**d**) or that contained two sgRNAs from different vectors or the same vector (**e**). **f**, Uniform Manifold Approximation and Project (UMAP) depicting the six clusters of CD8^+^ T cells obtained from graph-based clustering and *Tox* expression. **g**, Relative expression of CTL signature genes in the six clusters corresponding to Tpex (clusters 0, 1 and 2), Tex (clusters 3 and 4) and Teff (cluster 5) cells from (**f**). **h**, UMAP plots showing the expression of genes related to T cell exhaustion (including Tpex and Tex markers) or effector function in intratumoral OT-I cells as identified in scCRISPR screening. **i**, Activity scores of the curated Tpex-associated, Tex-associated and Teff-associated gene signatures among the Tpex, Tex and Teff cells from the scCRISPR experiment. **j**, Fold-change (FC)/FC plot comparing transcriptomic profiles of Tex relative to Tpex cells from our scCRISPR experiment (x-axis) with those from B16-OVA tumour-specific CD8^+^ T cells (y-axis). r, Pearson correlation coefficient. **k**, Relative intensity of peaks from upregulated open chromatin regions (OCRs) in intratumoral Tpex and Tex cells compared with splenic P14 cells from LCMV Armstrong infection (*n* = 3 per group). **l**, Geometric mean fluorescence intensity (gMFI) of TOX expression in OT-I cells from spleen or tdLN and Tpex and Tex OT-I cells from B16-OVA tumours (*n* = 10 per group). Naive endogenous splenic CD8^+^ T cells (*n* = 5). **m**, Frequencies of Tpex (Ly108^+^TIM-3^−^CD11c^−^), Tex (Ly108^−^TIM-3^+^CD11c^−^) and Teff (Ly108^−^TIM-3^+^CD11c^+^) cells in intratumoral OT-I cells in B16-OVA tumours on day 7 after adoptive transfer (upper). Lower, TOX or CD39 expression in indicated subsets (*n* = 5 per group). **n**, Relative activity scores of the gene programmes A–D (as indicated) in the six clusters from (**f**). **o**, Regulatory connections between the nine TF modules and four gene programmes. Red and blue lines indicate positive and negative regulatory effects, respectively. Line width shows regulation strength (see Methods for details). Data are representative of three independent experiments (**l**,**m**). ***P < 0.001; two-tailed Wilcoxon rank sum test (**i**), two-tailed unpaired Student’s *t*-test (**j**) or one-way ANOVA (**l**,**m**). Data are presented as the mean ± s.e.m. Source Data

To use scCRISPR screening for gene regulatory network (GRN) mapping, we re-engineered a dual-guide, direct-capture lentiviral single guide RNA (sgRNA) vector^11^ to generate a modified Ametrine-expressing retroviral vector that effectively transduced primary CD8^+^ T cells (Extended Data Fig. 1a,b). This was followed by the synthesis of a scCRISPR knockout (KO) library that targeted transcription factors (TFs), which are arguably the most potent regulators of cell fate decisions. To select these TFs, we performed computational analyses (differential expression, differential chromatin accessibility and TF motif enrichment) of four public RNA-seq and ATAC-seq datasets profiling CD8^+^ T cell subsets (early compared with late exhausted cells or T_pex_ cells compared with T_ex_ cells)^5,12–14^ (Extended Data Fig. 1c). The candidates enriched in at least two out of three analyses were compiled (Supplementary Table 1), and the final library targeted 180 curated TFs (in 360 dual-guide vectors) to ensure sufficient coverage for scCRISPR screening^15^ and non-targeting controls (NTCs) (Supplementary Table 2).

**Fig. 1:**
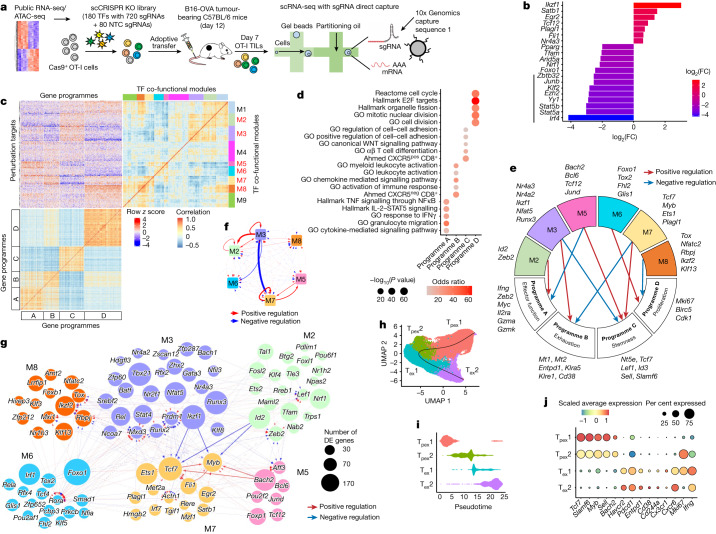
In vivo scCRISPR screening of intratumoral CTLs reveals connectivity of co-functional modules and gene programmes. **a**, Schematic of the scCRISPR screening strategy. **b**, Relative ratio (log_2_(fold-change (FC))) of cells with gene-level perturbation compared with sgNTC. Vertical line, TFs excluded for initial network analyses. **c**, Co-functional modules (with the six major modules highlighted in red) and co-regulated programmes (A–D) were identified by hierarchical clustering. **d**, Top enriched pathways (two-tailed Fisher’s exact test) in the four co-regulated gene programmes. **e**, Representation of regulatory connections between the six major modules and gene programmes from **c**. **f**, The interaction strengths between modules. Arrow width indicates interaction strength. **g**, The connectivity between the components of the indicated modules. Arrows indicate positive (red) and negative (blue) regulatory effects. Bold arrows highlight strong interactions between the indicated TFs. Node size, relative to number of perturbation-induced differentially expressed (DE) genes. **h**, UMAP showing the developmental trajectory of T_pex_1, T_pex_2, T_ex_1 and T_ex_2 cells among *Tox*^+^ cells. **i**, Pseudotime analysis of the indicated states from **h**. **j**, Relative expression of cell-state-associated genes.

Next, we transduced Cas9-expressing activated OT-I CD8^+^ T cells (specific for ovalbumin (OVA)) with the scCRISPR library, followed by adoptive transfer to B16-OVA melanoma tumour-bearing mice^16^. Single-cell sgRNA and transcriptome libraries from donor-derived tumour-infiltrating lymphocytes (TILs) were assessed by droplet-based sequencing 7 days later (Fig. 1a). We detected at least one sgRNA in the majority (82%) of cells, and about 81% of cells containing two sgRNAs contained ones from the same vector (Extended Data Fig. 1d,e). In the 42,209 cells bearing a single gene perturbation, we calculated the ratio of each genetic perturbation compared with the NTC, which revealed putative positive (*Stat5a*, *Stat5b* and *Irf4*) and negative (*Nr4a3* and *Fli1*) regulators of intratumoral CTL accumulation (Fig. 1b).

To interrogate cellular heterogeneity and the underlying transcriptional drivers, we visualized single-cell transcriptomes using uniform manifold approximation and projection (UMAP). Clusters 0–4 expressed *Tox*, a key regulator of exhaustion^17–21^. Within these, clusters 0−2 expressed the stemness-associated markers *Tcf7* (which encodes TCF-1), *Slamf6* (which encodes Ly108) and *Sell* (which encodes CD62L). Clusters 3 and 4 had abundant *Pdcd1* (which encodes PD-1) and *Havcr2* (which encodes TIM-3) levels, with cluster 4 showing the highest expression of the terminal exhaustion markers *Entpd1* (which encodes CD39), *Cd38* and *Cd244a* (Extended Data Fig. 1f–h). By contrast, cluster 5 (*Tox*^lo^*Entpd1*^lo^) expressed high levels of effector markers *Ifng*, *Gzma* and *Gzmb* (which encodes granzyme B (GZMB)) and *Itgax* (which encodes CD11c)^22^ (Extended Data Fig. 1f–h). Based on the expression of these markers^1,2^ and on T_pex_, T_ex_ and effector T (T_eff_) cell signatures (Extended Data Fig. 1i), we annotated these clusters as T_pex_ (*Tox*^+^*Tcf7*^+^*Havcr2*^–^), T_ex_ (*Tox*^+^*Tcf7*^–^*Havcr2*^+^) and T_eff_ cells (*Tox*^–^*Itgax*^+^*Havcr2*^+^) (Extended Data Fig. 1g). The differential gene expression profiles in T_ex_ compared with T_pex_ cells were highly correlated with a previous dataset^5^, and T_pex_ and T_ex_ cells showed increased chromatin accessibility of exhaustion-associated genes compared with T cells from acute lymphocytic choriomeningitis virus (LCMV) infection^23^ (Extended Data Fig. 1j,k). Finally, intratumoral T_pex_ and T_ex_ cells (among OT-I cells) displayed increased TOX expression compared with OT-I cells from the spleen and tumour-draining lymph node (tdLN) (Extended Data Fig. 1l). These results collectively provide support for their annotations as T_pex_ and T_ex_ cells. By contrast, T_eff_ cells showed reduced TOX and CD39 expression relative to T_ex_ cells and represented a minor population (Extended Data Fig. 1m), a result consistent with CTL adaptation to an exhausted state for better persistence in the TME^1,2^. Together, these scCRISPR screens in vivo and transcriptome analyses reveal molecular and cellular diversity in tumour-specific CTLs.

## Co-functional modules and gene programmes

To establish co-functional modules and downstream gene programmes, we first analysed differential gene expression patterns by comparing 172 TF perturbations (compared with NTC) with sufficient numbers of cells detected^15^. We then calculated the regulatory effects of each TF perturbation on target gene expression to identify co-functional TF modules based on their similar regulatory effects and to group target genes into co-regulated gene programmes^24^. We identified nine co-functional TF modules with convergent or divergent functional effects (Fig. 1c and Supplementary Table 3), and four co-regulated gene programmes associated with effector function (programme A), exhaustion (programme B), stemness (programme C) and proliferation (programme D) (Fig. 1c and Supplementary Table 4). These gene programmes showed distinct molecular signatures (Fig. 1d) and discrete enrichments in the T_pex_, T_ex_ and T_eff_ cell clusters (Extended Data Fig. 1n).

We next visualized the strength of perturbation effects of the nine co-functional modules on the four co-regulated gene programmes and identified six modules (M2, M3 and M5–M8) with marked effects (Fig. 1e and Extended Data Fig. 1o). The strongest negative and positive regulators of effector function programme were M5 (including *Bach2* and *Bcl6*) and M2 (*Id2* and *Zeb2*), respectively. The strongest negative and positive regulators of the exhaustion programme were M7 (*Tcf7*, *Myb* and *Ets1*) and M3 (*Nr4a2*, *Nr4a3* and *Ikzf1* (which encodes IKAROS)), respectively, whereas the stemness programme was boosted by M7 and suppressed by M3. This result suggests that there is reciprocal regulation of exhaustion and stemness programmes by these two modules. M5 was another notable positive regulator for the stemness programme. Finally, the top negative and positive regulators of proliferation programme were M8 (*Tox* and *Rbpj*) and M6 (*Foxo1*), respectively (Fig. 1e). These results demonstrate the complex but concerted effects of these modules on effector function, exhaustion, stemness and proliferation programmes.

To uncover intramodular and intermodular regulatory circuits, we generated a focused GRN between the six main modules and assessed the interaction strengths (Fig. 1f). Strong positive intramodular interactions within M3 and M7 were observed. There were also mutual positive intermodular interactions between the stemness-promoting M5 and M7 programmes, and between the exhaustion-promoting M2 and M3 programmes (Fig. 1f and Extended Data Fig. 1o), which suggested that there was intermodular self-reinforcements of stemness and exhaustion. Conversely, the negative effect imposed by M3 on M7 suggested that inhibition of stemness by the exhaustion programme may potentiate terminal differentiation (Fig. 1f). To uncover specific regulation between individual TFs, we first constructed connectivity maps between TFs within and across the modules and then defined central hub TFs. *Rbpj*, *Ikzf2* and *Klf13* (M8), *Runx3*, *Ikzf1* and *Nfat5* (M3), *Foxo1* (M6), *Tcf7*, *Myb* and *Ets1* (M7), *Bach2* (M5), and *Id2* (M2) had large regulatory effects in their respective modules (Fig. 1g), thereby identifying them as central hub TFs. Beyond capturing known interactions (for example, *Tcf7* (ref. ^3^), *Bach2* (ref. ^14^) and *Myb*^25^), this analysis revealed many previously uncharacterized interactions (Fig. 1g and Supplementary Table 5). Collectively, we revealed intramodular and intermodular regulatory circuits and central hub TFs that probably underlie intratumoral CTL responses.

## State-specific transcriptional drivers

**Extended Data Fig. 2:**
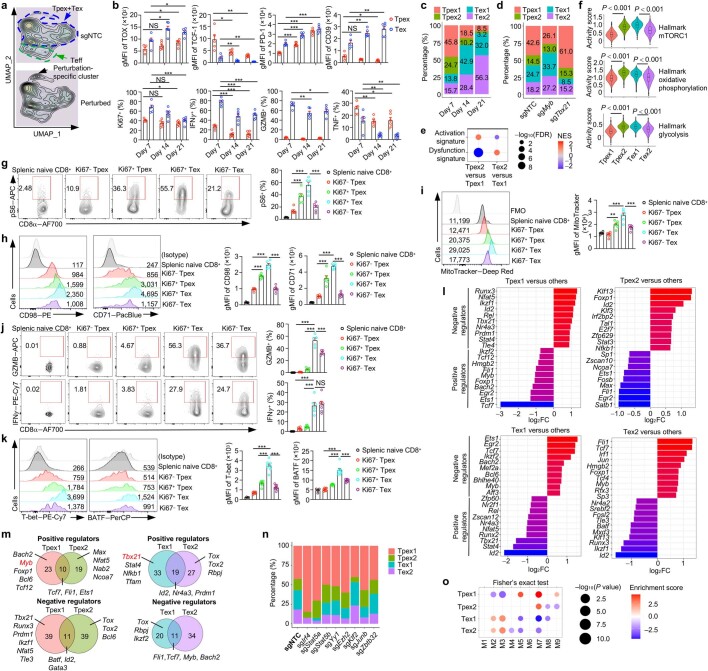
Characterization of CTL differentiation states and identification of underlying drivers by scCRISPR screening. **a**, UMAP plots with arrows depicting the combined Tpex and Tex (Tpex + Tex) and Teff cluster distribution, and the perturbation-specific cluster in intratumoral OT-I cells from scCRISPR screening. **b**, TOX, TCF-1, PD-1, and CD39 expression in intratumoral Tpex or Tex OT-I cells, or frequencies of Ki67^+^, IFNγ^+^, GZMB^+^ or TNF^+^ cells among Tpex or Tex OT-I cells on days 7 (*n* = 5), 14 (*n* = 6) or 21 (*n* = 6) after adoptive transfer to B16-OVA tumour-bearing mice. **c**, Percentages of indicated OT-I cell states on days 7, 14 or 21 as in (**b**) as assessed by scRNA-seq. **d**, Percentages of OT-I cell states in cells transduced with sgNTC, sg*Myb* and sg*Tbx21* from scCRISPR screening. **e**, Pre-ranked GSEA analysis of Tpex2 compared with Tpex1 and Tex2 compared with Tex1 cells using the indicated gene signatures. NES, normalized enrichment score. **f**, Activity scores of indicated gene signatures in OT-I cell states, and *n* = 6,202 (Tpex1), *n* = 3,246 (Tpex2), *n* = 7,695 (Tex1) or *n* = 8,631 (Tex2) cells examined over one independent experiment. The boxes stand for 25% to 75% interquartile range (IQR), and the whiskers stand for minimum (25% quantile – 1.5* IQR) to maximum (75% quantile + 1.5* IQR) values. **g**–**k**, Analyses of indicated intraumoral Tpex or Tex populations or splenic naive CD8^+^ T cells. Frequency of pS6^+^ cells (*n* = 5 for splenic naive CD8^+^ and 6 for Ki67^–^ Tpex, Ki67^+^ Tpex, Ki67^+^ Tex, and Ki67^–^ Tex) (**g**). CD98 and CD71 expression (*n* = 6 per group) (**h**). MitoTracker levels in cells (*n* = 6 per group) (**i**). Frequencies of GZMB^+^ (*n* = 5 per group) and IFNγ^+^ (*n* = 6 per group) cells (**j**). T-bet and BATF expression (*n* = 4 for splenic naive CD8^+^ and 8 for Ki67^–^ Tpex, Ki67^+^ Tpex, Ki67^+^ Tex, and Ki67^–^ Tex) (**k**). FMO, Fluorescence Minus One. **l**, Top positive and negative regulators of each individual cell state (compared with all other states) in scCRISPR screening. **m**, Venn diagrams depicting the positive and negative transcriptional regulators of Tpex1 compared with Tpex2 or Tex1 compared with Tex2 states based on scCRISPR screening, with their discrete and overlapping distributions shown. **n**, Percentages of indicated cell states among cells with indicated perturbations in scCRISPR screening. The same sgNTC cells are presented in (**d**). **o**, Enrichment scores (colour-coded) of a co-functional module as a positive or negative regulator of the indicated cell states. Circle size indicates significance (*P* < 0.05). Data are representative of one (**b**), three (**g**,**h**,**j**,**k**) or two (**i**) independent experiments. NS, not significant; *P < 0.05, **P < 0.01, and ***P < 0.001; two-tailed unpaired Student’s *t*-test (**b**), two-tailed Wilcoxon rank sum test (**f**), one-way ANOVA (**g**–**k**) or right-tailed Fisher’s exact test (**o**). Data are presented as the mean ± s.e.m. Source Data

As TF perturbations may exert regulatory effects on gene programmes by inducing cell population changes, we examined perturbation effects on intratumoral CTL heterogeneity, focusing on T_pex_ and T_ex_ cell populations. A perturbation-only population that did not contain cells expressing NTC sgRNAs (sgNTCs) was identified and resembled T_pex_ cells (cluster 0; Extended Data Fig. 1g), whereas the remaining clusters contained both sgNTC and perturbation sgRNA-transduced cells (Extended Data Fig. 2a). Discrete T_pex_-associated and T_ex_-associated markers^5^ and their progressive changes^13^ were dynamically regulated during tumour development (Extended Data Fig. 2b). Given the identification of intermediate and transitory T_ex_ cells in chronic infection^26,27^, we operationally classified T_pex_ and T_ex_ clusters as precursor exhausted-like state 1 (T_pex_1), T_pex_2, terminal exhausted-like state 1 (T_ex_1) and T_ex_2 cells, with pseudotime analysis predicting a trajectory from T_pex_1, through transitional T_pex_2 and T_ex_1 cell states, to T_ex_2 cells (Fig. 1h,i). Accordingly, the T_ex_2 but not the T_ex_1 cell proportion continuously increased, whereas the two T_pex_ cell states decreased during tumour progression (Extended Data Fig. 2c). Finally, as expected, T_pex_1 and T_ex_1 cells were reduced following perturbation of *Myb*^25^ and *Tbx21* (which encodes T-bet)^26,28^, respectively (Extended Data Fig. 2d). These results provide further support for these annotations.

Gene set enrichment analysis (GSEA) revealed an increased activation-specific signature^29^ in T_pex_2 compared with T_pex_1 cells and a dysfunction-associated signature^29^ in T_ex_2 compared with T_ex_1 cells (Extended Data Fig. 2e). Furthermore, the T_pex_1 cell state expressed stemness-associated genes that were progressively downregulated during differentiation (Fig. 1j). Conversely, the T_pex_2 cell state expressed *Ifng* and the proliferative marker *Mki67* and had higher activities of mTORC1-associated and metabolism-associated signatures than the T_pex_1 cell state (Fig. 1j and Extended Data Fig. 2f). This result indicated their exit from a stem-like, quiescent state that is associated with metabolic reprogramming^30^. Furthermore, T_ex_1 cells retained high *Mki67* expression and, compared with T_ex_2 cells, showed higher metabolic signatures but lower levels of terminal exhaustion markers^13^ (Fig. 1j and Extended Data Fig. 2f), which made them partially resemble intermediate T_ex_ cells^26,27^. Accordingly, Ki67^+^ T_pex_ cells and Ki67^+^ T_ex_ cells (corresponding to the T_pex_2 cell state and T_ex_1 cell state, respectively) had higher mTORC1 activity (based on phosphorylated S6 (pS6), CD98, CD71 and MitoTracker staining)^30^ compared with their Ki67^–^ counterparts (T_pex_1 and T_ex_2 cell states) (Extended Data Fig. 2g–i). Ki67^+^ T_ex_ cells also expressed the highest levels of GZMB, T-bet and BATF and comparable IFNγ levels to Ki67^–^ T_ex_ cells (Extended Data Fig. 2j,k), which indicated that these cells have a strong effector function.

We next identified transcriptional activators and repressors for each cell state based on sgRNA-mediated depletion or enrichment (compared with the other three counterparts) (Extended Data Fig. 2l and Supplementary Table 6). This analysis also revealed shared and selective (for example, *Myb*^25^ and *Tbx21*^26,28^) regulators for each state (Extended Data Fig. 2m). Furthermore, visualization of the perturbation effects after targeting the eight TFs (Fig. 1b) excluded from the abovementioned transcriptome analysis revealed their effects on cell states, including reduced T_ex_ cell percentages after targeting *Stat5a*, *Stat5b* and *Irf4* (Extended Data Fig. 2n). Altogether, analyses of state-specific regulators identified transcriptional drivers that mediate CTL heterogeneity.

To determine the extent to which CTL differentiation states are shaped by the co-functional modules, we examined whether a module was enriched among the top regulated genes within each state. M7 was enriched as a positive and negative regulator of T_pex_ and T_ex_ cell states, respectively (Extended Data Fig. 2o), a finding consistent with its stemness-promoting effects (Fig. 1e). Conversely, M3 and, to a lesser extent, M2 were negative regulators of T_pex_1 but positive regulators of T_ex_ cells (Extended Data Fig. 2o), a result also consistent with their effects on gene programmes (Extended Data Fig. 1o). Collectively, these results reveal state-specific transcriptional drivers and co-functional modules that underlie progressive CTL differentiation.

## The IKAROS–TCF-1 axis in T_pex_ cell quiescence exit

**Fig. 2:**
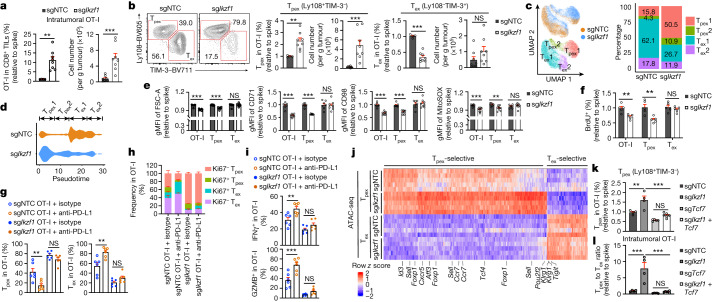
IKAROS promotes the quiescence exit of T_pex_1 cells. **a**,**b**, sgNTC (*n* = 4) or sg*Ikzf1* (*n* = 8) OT-I cells were co-transferred with sgNTC-expressing (spike) cells (dual-colour transfer system) into B16-OVA tumour-bearing mice. **a**, Relative frequency (normalized to spike) and number (per g of tumour tissue) of OT-I cells. **b**, Frequencies and numbers of T_pex_ and T_ex_ OT-I cells. **c**, scRNA-seq analysis of sgNTC and sg*Ikzf1* OT-I cells and cell cluster proportions from B16-OVA tumours. **d**, Pseudotime analysis of cell states from **c**. **e**,**f**, Relative (normalized to spike) geometric mean fluorescence intensities (gMFIs) of indicated markers (**e**) or relative frequency of BrdU^+^ (**f**) cells (dual-colour transfer system in B16-OVA tumours) (*n* = 7 for sgNTC and *n* = 8 for sg*Ikzf1* in **e**; 5 per group in **f**). **g**–**i**, B16-OVA tumour-bearing mice that received sgNTC or sg*Ikzf1* OT-I cells were treated with anti-PD-L1 or isotype control (*n* = 6 per group). Frequencies of indicated subsets (**g**), Ki67^–^ T_pex_, Ki67^+^ T_pex_, Ki67^+^ T_ex_ and Ki67^–^ T_ex_ cells (**h**), or IFNγ^+^ and GZMB^+^ OT-I cells (**i**). **j**, T_pex_-selectively and T_ex_-selectively accessible peaks in ATAC-seq analysis of sgNTC and sg*Ikzf1* T_pex_ and T_ex_ cells (*n* = 5 per group). **k**,**l**, Relative frequency of T_pex_ cells (**k**) or T_pex_ to T_ex_ cell ratio (**l**) of sgNTC (*n* = 4), sg*Ikzf1* (*n* = 4), sg*Tcf7* (*n* = 5) or sg*Ikzf1* with sg*Tcf7* (*n* = 6) OT-I cells (dual-colour transfer system in B16-OVA tumours). Data are representative of three (**a**,**b**,**e**), two (**f**,**k**,**l**) or one (**g**–**i**) independent experiments. NS, not significant, **P < 0.01, ***P < 0.001; two-tailed unpaired Student’s *t*-test (**a**,**b**,**e**,**f**) or one-way analysis of variance (ANOVA) (**g**,**i**,**k**,**l**). Data are presented as the mean ± s.e.m. Source Data

**Extended Data Fig. 3:**
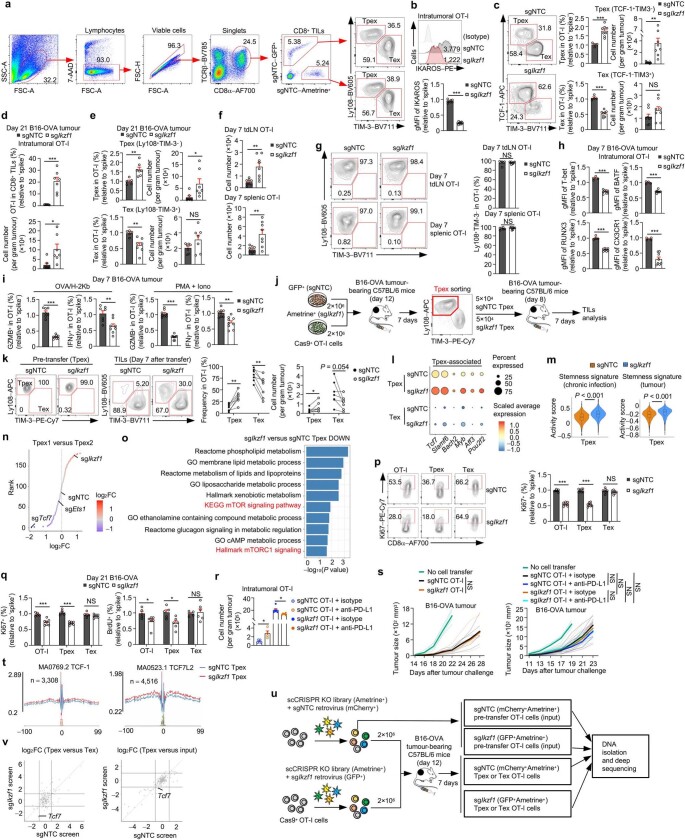
*Ikzf1* deficiency promotes Tpex1 cell accumulation. **a**, Gating strategies of flow cytometry analysis to identify the sgNTC OT-I cells labeled with different fluorescent proteins and their Tpex and Tex subsets in the dual-colour transfer system. **b**, IKAROS expression in total intratumoral sgNTC (*n* = 8) or sg*Ikzf1* (*n* = 9) OT-I cells (dual-colour transfer system). **c**, Relative frequencies and numbers of sgNTC (*n* = 4) and sg*Ikzf1* (*n* = 8) Tpex (TCF-1^+^TIM-3^–^) and Tex (TCF-1^–^TIM-3^+^) OT-I cells (dual-colour transfer system). **d**,**e**, Relative frequencies and numbers of total (**d**), Tpex or Tex (**e**) sgRNA OT-I cells (dual-colour transfer system) from B16-OVA tumours were analyzed on day 21 after adoptive transfer (*n* = 5 for sgNTC and *n* = 7 for sg*Ikzf1*). **f**, Numbers of OT-I cells in tdLN and spleen (dual-colour transfer system) on day 7 after adoptive transfer (*n* = 8 per group). **g**, Relative frequencies of Ly108^+^TIM-3^–^ cells among tdLN and splenic OT-I cells (dual colour-transfer system) (*n* = 6 for sgNTC and *n* = 8 for sg*Ikzf1*). **h**, T-bet, BATF, RUNX3 and CX3CR1 expression in intratumoral OT-I cells (dual-colour transfer system) (*n* = 4 for sgNTC and *n* = 8 for sg*Ikzf1*). **i**, Relative frequencies of GZMB^+^ and IFNγ^+^ OT-I cells (dual-colour transfer system) after cognate antigen (*n* = 7 for sgNTC and *n* = 8 for sg*Ikzf1*) or PMA plus ionomycin (PMA + Iono) (*n* = 8 for sgNTC and *n* = 9 for sg*Ikzf1*) stimulation ex vivo. **j**,**k**, Schematic for secondary Tpex cell transfer assay (**j**). Frequencies and numbers of Tpex and Tex cells from Tpex secondary transfer assay (**k**) (*n* = 7 per group). **l**, Relative average expression of the selected genes in indicated Tpex and Tex OT-I cells as profiled by scRNA-seq (see Fig. 2c). **m**, Activity scores of stemness-associated signatures (see Methods) in the sgNTC or sg*Ikzf1* Tpex OT-I cell subset from Fig. 2c, and *n* = 2,825 (sgNTC) or *n* = 9,151 (sg*Ikzf1*) cells examined over one independent experiment. The boxes stand for 25% to 75% interquartile range (IQR), and the whiskers stand for minimum (25% quantile – 1.5* IQR) to maximum (75% quantile + 1.5* IQR) values. **n**, Top enriched (red) and depleted (blue) perturbations in Tpex1 compared with Tpex2 cells. **o**, Pathway enrichment analysis revealing enrichment of metabolic pathway-related signatures among downregulated (DOWN) genes in sg*Ikzf1* Tpex cells compared with sgNTC Tpex cells. **p**, Relative frequencies of Ki67^+^ cells among total sgNTC (*n* = 7) or sg*Ikzf1* (*n* = 8) intratumoral OT-I cells and their Tpex and Tex subsets (dual-colour transfer system) on day 7 after adoptive transfer. **q**, Relative frequencies of Ki67^+^ (*n* = 5 for sgNTC and *n* = 7 for sg*Ikzf1*) and BrdU^+^ (*n *= 5 for sgNTC and *n* = 6 for sg*Ikzf1*) cells among indicated sgRNA-transduced intratumoral OT-I cell populations (dual-colour transfer system) on day 21 after adoptive transfer. **r**, Numbers of total intratumoral OT-I cells in mice given the indicated treatments (*n* = 5 for sgNTC OT-I + isotype, *n* = 4 for sgNTC OT-I + anti-PD-L1, and *n* = 6 for sg*Ikzf1* OT-I + isotype, sg*Ikzf1* OT-I + anti-PD-L1). **s**, Left, B16-OVA tumour growth in mice that received sgNTC (*n* = 7) or sg*Ikzf1* (*n* = 6) OT-I cells. No cell transfer group (*n* = 4). Right, B16-OVA tumour growth in mice given the indicated treatments (*n* = 4 for no cell transfer, *n* = 8 for sgNTC OT-I + isotype, *n* = 7 for sgNTC OT-I + anti-PD-L1 and *n* = 9 for sg*Ikzf1* OT-I + isotype, sg*Ikzf1* OT-I + anti-PD-L1). **t**, Footprinting analysis of TCF-1 and TCF7L2 in ATAC-seq analysis of sgNTC and sg*Ikzf1* Tpex OT-I cells (*n* = 5 per group). **u**, **v**, Schematic for genetic interaction screening of sgRNA-transduced OT-I cells (e.g., sgNTC or sg*Ikzf1*) (see Methods) (**u**). Sectored scatter plots of gene-level log_2_FC from sgNTC (x-axis) and sg*Ikzf1* (y-axis) OT-I cells in genetic interaction screening. The intratumoral Tpex compared with Tex cells and Tpex cells compared with input are shown, with *Tcf7* highlighted (**v**). Data are representative of three (**b**,**c**,**f**–**h,p**), one (**d**,**e**,**q**,**r**) or two (**i**,**k**,**s**) independent experiments. NS, not significant; *P < 0.05, **P < 0.01, and ***P < 0.001; two-taiIed unpaired Student’s *t*-test (**b**–**i**,**p**,**q**), two-tailed paired Student’s *t*-test (**k**), two-tailed Wilcoxon rank sum test (**m**), two-tailed Fisher’s exact test (**o**), one-way ANOVA (**r**) or two-way ANOVA (**s**). Data are presented as the mean ± s.e.m. Source Data

Targeting *Ikzf1* (from M3) resulted in the strongest accumulation of intratumoral CTLs (Fig. 1b). To explore cell-intrinsic roles of *Ikzf1*, we used a dual-colour transfer system^16,23^, wherein the use of different fluorescent proteins did not alter CTL responses (Extended Data Fig. 3a). OT-I cells expressing *Ikzf1* sgRNA (sg*Ikzf1*) showed efficient *Ikzf1* gene targeting (Extended Data Fig. 3b and Supplementary Table 7) and were markedly accumulated in the TME at day 7 after transfer (Fig. 2a). T_pex_ cells increased after *Ikzf1* perturbation, whereas the percentage, but not the number, of T_ex_ cells was reduced (Fig. 2b and Extended Data Fig. 3c). *Ikzf1* deficiency exerted similar effects at day 21 after transfer (Extended Data Fig. 3d,e). Besides the TME, sg*Ikzf1* OT-I cells (mainly Ly108^+^TIM-3^–^) accumulated in the tdLN and spleen (Extended Data Fig. 3f,g). Notably, sg*Ikzf1* intratumoral OT-I cells had reduced expression of effector and cytotoxic molecules (Extended Data Fig. 3h,i). To determine the role of *Ikzf1* in the T_pex_ to T_ex_ cell transition, we sorted T_pex_ cells targeted with sgNTC or sg*Ikzf1* from B16-OVA tumours and transferred them to new tumour-bearing mice^5,9^ (Extended Data Fig. 3j). In this secondary transfer assay, *Ikzf1* deficiency was associated with the accumulation of T_pex_ cells and a reduction in T_ex_ cells (Extended Data Fig. 3k). Thus, IKAROS promotes T_pex_ to T_ex_ cell differentiation.

To establish the effect of *Ikzf1* deficiency on CTL heterogeneity in an unbiased manner, we performed scRNA-seq analysis. sg*Ikzf1* OT-I cells were transcriptionally distinct from sgNTC OT-I cells and contained more T_pex_ cells, especially T_pex_1 cells, but fewer T_ex_ cells (Fig. 2c). sg*Ikzf1* T_pex_ cells also upregulated stemness-associated TFs^14^ and gene signatures^7,20^ (Extended Data Fig. 3l,m). Pseudotime analysis indicated that sg*Ikzf1* cells mainly accumulated in the T_pex_1 cell state (Fig. 2d), a finding supported by sg*Ikzf1* enrichment among the top-most perturbations affecting the T_pex_1 to T_pex_2 cell ratio (Extended Data Fig. 3n and Supplementary Table 8). Moreover, sg*Ikzf1* T_pex_ cells downregulated multiple metabolic and mTORC1 signatures^30^ (Extended Data Fig. 3o), which raised the possibility of aberrant metabolic quiescence^30^. Indeed, sg*Ikzf1* T_pex_ cells showed reduced mTORC1-associated features and reduced levels of MitoSOX and proliferation markers (Ki67 and bromodeoxyuridine (BrdU)) at day 7 after transfer (Fig. 2e,f and Extended Data Fig. 3p), with such proliferative defects also evident at day 21 (Extended Data Fig. 3q). Thus, targeting *Ikzf1* inhibits the T_pex_1 to T_pex_2 cell transition and associated metabolic rewiring and quiescence exit^26,30^.

As ICB induces differentiation of T_pex_ cells into T_ex_ cells^5,7,26,27,31^, we tested the effect of *Ikzf1* deficiency on ICB responses by treating tumour-bearing mice that received sgNTC or sg*Ikzf1* OT-I cells with anti-PD-L1. Unlike sgNTC OT-I cells, sg*Ikzf1* OT-I cells did not increase after anti-PD-L1 treatment or display altered differentiation states (Fig. 2g,h and Extended Data Fig. 3r). sg*Ikzf1* cells also did not upregulate IFNγ or GZMB expression after anti-PD-L1 treatment (Fig. 2i). Moreover, tumour sizes were comparable in mice that received transfer of sg*Ikzf1* or sgNTC OT-I cells alone or in combination with anti-PD-L1 (Extended Data Fig. 3s). Thus, despite their increased accumulation, sg*Ikzf1* cells do not gain added antitumour effects, which is probably due to their aberrant quiescence state and failure to differentiate into T_ex_ cells.

To gain additional mechanistic insights, we performed ATAC-seq analysis. sg*Ikzf1* T_pex_ cells showed increased accessibility of T_pex_-selective open chromatin regions (OCRs) but reduced accessibility of T_ex_-selective OCRs (Fig. 2j), which indicated an enhanced stemness-associated and reduced exhaustion-associated epigenetic programme. TF footprinting analysis predicted increased binding activity of stemness-associated TCF/LEF family members in sg*Ikzf1* T_pex_ cells (Extended Data Fig. 3t). To identify IKAROS downstream targets in a more unbiased manner, we performed genetic interaction screens in vivo^10^ by transducing OT-I cells expressing sgNTC or sg*Ikzf1* together with the abovementioned TF sgRNA library, followed by transfer to tumour-bearing mice (Extended Data Fig. 3u). We nominated functionally relevant targets of IKAROS by identifying perturbations that reversed the T_pex_ to T_ex_ cell ratio and T_pex_ cell accumulation (Supplementary Table 9), and found that *Tcf7* co-targeting blocked both of these parameters in sg*Ikzf1* cells (Extended Data Fig. 3v). Accordingly, our validation experiments showed that co-targeting *Ikzf1* and *Tcf7* rectified the alterations in T_pex_ cells and the T_pex_ to T_ex_ cell ratio (Fig. 2k,l) observed in *Ikzf1*-deficient cells. These results indicate that IKAROS affects T_pex_ to T_ex_ cell differentiation largely by restraining TCF-1.

## The ETS1–BATF axis limits T_ex_1 cell generation

**Fig. 3:**
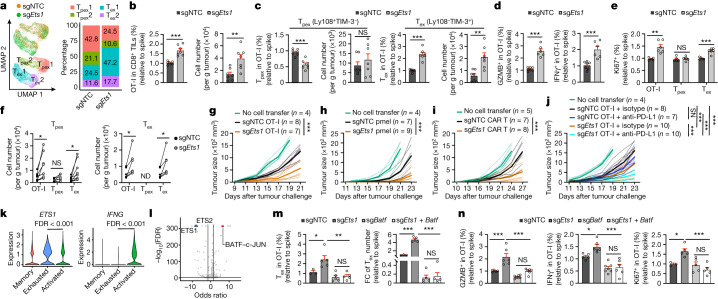
ETS1 is a gatekeeper for the T_pex_ to T_ex_1 cell transition. **a**, sgNTC and sg*Ets1* OT-I cells and cell cluster proportions. **b**,**c**, Relative frequencies and numbers of total intratumoral OT-I cells (**b**) and their T_pex_ and T_ex_ cell subsets in B16-OVA tumours on day 7 (**c**) (*n* = 7 per group). **d**,**e**, Relative frequencies of GZMB^+^ and IFNγ^+^ (*n* = 7 for sgNTC and 6 for sg*Ets1*) OT-I cells after OVA/H-2Kb stimulation (**d**) or Ki67^+^ OT-I populations (*n* = 5 for sgNTC and 6 for sg*Ets1*) (**e**). **f**, Numbers of total, T_pex_ and T_ex_ OT-I cells after T_pex_ (left, *n* = 9 per group) or T_ex_ (right, *n* = 6 per group) cell secondary transfer. ND, not detected. **g**, B16-OVA tumour growth with sgNTC or sg*Ets1* OT-I cell treatment. **h**, B16-F10 tumour growth with sgNTC or sg*Ets1* pmel cell treatment. **i**, B16-hCD19 tumour growth with sgNTC or sg*Ets1* hCD19 CAR T cell treatment. **j**, B16-OVA tumour growth with indicated treatments. **k,** *ETS1* and *IFNG* expression in memory, exhausted and activated CD8^+^ T cells from patients with BCC (data from Gene Expression Omnibus (GEO) database identifier GSE123813). FDR, false discovery rate. **l**, TF motif enrichment analysis in sg*Ets1* compared with sgNTC T_ex _cells (*n* = 4 per group). **m**, Relative frequency and number of sgNTC (*n* = 3), sg*Ets1* (*n* = 5), sg*Batf* (*n* = 5), or sg*Ets1* with sg*Batf* (*n* = 5) T_ex_ cells. **n**, Relative frequencies of GZMB^+^, IFNγ^+^ (*n* = 5 for sgNTC, *n* = 6 for sg*Ets1* and sg*Ets1* with sg*Batf*, and *n* = 7 for sg*Batf*) and Ki67^+^ (*n* = 3 for sgNTC, and *n* = 5 for sg*Ets1*, sg*Batf*, and sg*Ets1* with sg*Batf*) intratumoral OT-I cells. Data are representative of three (**b**–**e**,**g**), two (**f**,**h**,**i**,**m**,**n**) or one (**j**) independent experiments. *P < 0.05, **P < 0.01, ***P < 0.001; two-tailed unpaired Student’s *t*-test (**b**–**e**), two-tailed paired Student’s *t*-test (**f**), two-way ANOVA (**g**–**j**), two-tailed Wilcoxon rank-sum test (**k**) or one-way ANOVA (**m**,**n**). Data are presented as the mean ± s.e.m. Source Data

**Extended Data Fig. 4:**
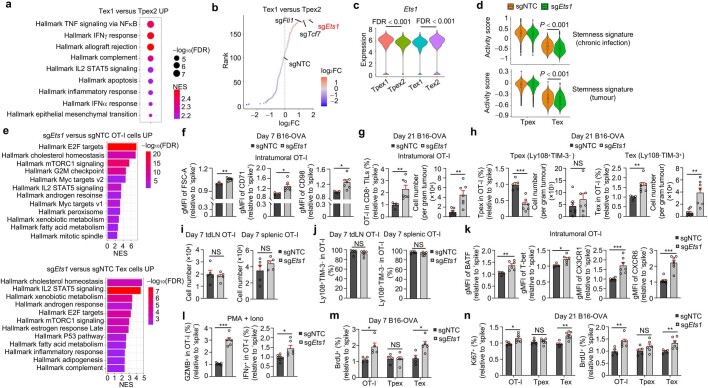
Targeting *Ets1* promotes Tex1 cell accumulation and antitumour immunity. **a**, GSEA analysis revealing enrichment of indicated signatures among genes upregulated (UP) in Tex1 compared with Tpex2 cells as profiled by scCRISPR screening. **b**, Top enriched (red) and depleted (blue) perturbations in Tex1 compared with Tpex2 cells. **c**, Expression of *Ets1* in indicated OT-I cell states. **d**, Activity scores of the curated stemness-associated signatures in sgNTC and sg*Ets1* Tpex and Tex OT-I cells (from scRNA-seq analysis shown in Fig. 3a). **e**, GSEA analysis revealing enrichment of indicated signatures in sg*Ets1* compared with sgNTC total (upper) or Tex (lower) OT-I cells. **f**, Relative expression of FSC-A, CD71 and CD98 in intratumoral OT-I cells (dual-colour transfer system) (*n* = 3 for sgNTC and *n* = 7 for sg*Ets1*). **g**,**h**, Relative frequencies and numbers of total (**g**), Tpex (**h**) and Tex (**h**) OT-I cells in B16-OVA tumours on day 21 after adoptive transfer (*n* = 5 for sgNTC and *n* = 7 for sg*Ets1*). The same sgNTC OT-I cells are presented in Extended Data Fig. 3d, e. **i**, Numbers of OT-I cells in tdLN (left) and spleen (right) on day 7 after adoptive transfer (*n* = 5 per group). **j**, Frequencies of Ly108^+^TIM-3^–^ OT-I cells in tdLN and spleen (*n* = 6 per group). The same sgNTC OT-I cells are presented in Extended Data Fig. 3g. **k**, Relative expression of BATF, T-bet (*n* = 5 for sgNTC and *n* = 6 for sg*Ets1* for both BATF and T-bet), CX3CR1 (*n* = 7 per group) and CXCR6 (*n* = 7 per group) in intratumoral OT-I cells (dual-colour transfer system). **l**, Relative frequencies of GZMB^+^ and IFNγ^+^ OT-I cells (dual-colour transfer system) after PMA + Iono stimulation (*n* = 7 for sgNTC and *n* = 6 for sg*Ets1*). **m**, Relative frequencies of BrdU^+^ cells among indicated intratumoral OT-I cell populations (dual-colour transfer system) (*n* = 4 for sgNTC and *n* = 5 for sg*Ets1*) on day 7 after adoptive transfer. **n**, Relative frequencies of Ki67^+^ (*n* = 5 for sgNTC and *n* = 7 for sg*Ets1*; left) and BrdU^+^ (*n* = 5 for sgNTC and *n* = 6 for sg*Ets1*; right) cells among intratumoral OT-I cell populations (dual-colour transfer system) on day 21 after adoptive transfer. The same sgNTC OT-I cells are presented in Extended Data Fig. 3q. Data are representative of three (**f**,**i**,**j**–**l**), one (**g**,**h**,**n**) or two (**m**) independent experiments. NS, not significant; *P < 0.05, **P < 0.01, and ***P < 0.001; two-tailed Kolmogorov–Smirnov test with FDR adjustments for multiple comparisons (**a**,**e**), two-tailed Wilcoxon rank sum test (**c**,**d**) or two-tailed unpaired Student’s *t*-test (**f**–**n**). Data are presented as the mean ± s.e.m. Source Data

T_ex_1 cells showed increased effector-function-associated pathways compared with T_pex_2 cells (Extended Data Fig. 4a). We therefore focused on putative TFs that mediate the T_pex_2 to T_ex_1 cell transition and identified *Ets1* (from M7) as one of the top negative regulators (Extended Data Fig. 4b and Supplementary Table 8). Additionally, *Ets1* expression was downregulated in T_pex_2 and T_ex_1 cell states (Extended Data Fig. 4c). To examine the role of ETS1 in CTL heterogeneity, we effectively targeted *Ets1* in OT-I cells (Supplementary Table 7) and performed scRNA-seq. *Ets1*-deficient cells showed expansion of T_ex_1 cells, which was accompanied by a reduction in T_pex_ cell proportion (Fig. 3a) and stemness-associated signatures in T_ex_ cells (Extended Data Fig. 4d). Targeting *Ets1* also upregulated metabolic gene signatures and mTORC1-associated features (Extended Data Fig. 4e,f), which indicated an inhibitory effect of ETS1 on mTORC1 signalling.

**Extended Data Fig. 5:**
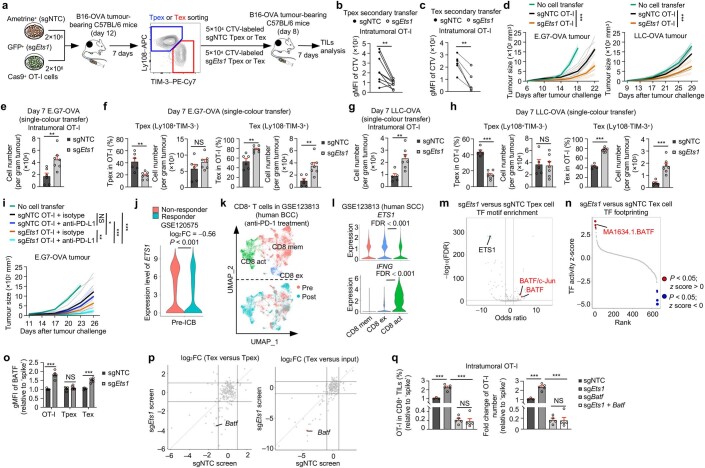
ETS1–BATF axis impinges upon Tpex to Tex cell transition. **a**–**c**, Schematic of Tpex or Tex cell secondary transfer assay (**a**). CellTrace Violet (CTV) levels in total intratumoral OT-I cells after Tpex (**b**) or Tex (**c**) cell secondary transfer (*n *= 9 per group for Tpex cells and *n* = 6 per group for Tex cells). **d**, Left, E.G7-OVA tumour growth in mice given sgNTC (*n* = 8) or sg*Ets1* (*n *= 9) OT-I cells. No cell transfer (*n* = 4). Right, LLC-OVA tumour growth in mice given sgNTC (*n* = 9) or sg*Ets1* (*n* = 10) OT-I cells. No cell transfer (*n* = 4). **e**,**f**, sgNTC (*n* = 6) or sg*Ets1* (*n* = 7) OT-I cells were transferred to E.G7-OVA tumour-bearing mice (single-colour transfer system). Number of total intratumoral OT-I cells (**e**). Frequencies and numbers of Tpex and Tex OT-I cells (**f**). **g**,**h**, sgNTC (*n* = 5) or sg*Ets1* (*n* = 7) OT-I cells were transferred to LLC-OVA tumour-bearing mice (single-colour transfer system). Number of total intratumoral OT-I cells (**g**). Frequencies and numbers of Tpex and Tex OT-I cells (**h**). **i**, E.G7-OVA tumour growth in mice given the indicated treatments (*n* = 3 for no cell transfer and 7 for all other groups). The same sgNTC OT-I + isotype and sgNTC + anti-PD-L1 groups are presented in Fig. 5h. **j**, *ETS1* expression in human intratumoral CD8^+^ T cells before ICB (Pre-ICB) treatment in individuals with melanoma. **k**, scRNA-seq analysis of T cells from patients with basal cell carcinoma (BCC) pre- and post-anti-PD-1 treatments. UMAP shows three CD8^+^ T cell subsets (memory (mem), exhausted (ex) and activated (act)) and the distribution of CD8^+^ T cells pre- or post-anti-PD-1 treatment. **l**, *ETS1* and *IFNG* expression in human CD8^+^ T cell subsets from scRNA-seq analysis of T cells from patients with squamous cell carcinoma (SCC) pre- and post-anti-PD-1 treatments. **m**, TF motif enrichment analysis of differentially accessible chromatin regions in sg*Ets1* compared with sgNTC Tpex cells by ATAC-seq (*n* = 4 per group). **n**, Footprinting analysis of ATAC-seq peaks in sgNTC and sg*Ets1* Tex cells, ranked by activity *z* scores. **o**, BATF expression in indicated intratumoral OT-I cell populations (dual-colour transfer system) (*n* = 5 for sgNTC and *n* = 6 for sg*Ets1*). **p**, Genetic interaction screen of sg*Ets1*-transduced and sgNTC-transduced OT-I cells (see Methods and similar schematic in Extended Data Fig. 3u). Sectored scatter plot of gene-level log_2_FC from sgNTC (x-axis) and sg*Ets1* (y-axis) OT-I cells in genetic interaction screening. The intratumoral Tex compared with Tpex cells and Tex cells compared with input are shown, with *Batf* highlighted. **q**, Relative frequency and fold change of number of cells expressing sgNTC (*n* = 3), sg*Ets1* (*n* = 5), sg*Batf* (*n* = 5) or sg*Ets1* with sg*Batf* (*n* = 5) (dual-colour transfer system). Data are representative of two (**b**–**d**,**q**), one (**e**–**i**) or three (**o**) independent experiments. NS, not significant; *P < 0.05, **P < 0.01 and ***P < 0.001; two-tailed paired Student’s *t*-test (**b**,**c**), two-way ANOVA (**d**,**i**), two-tailed unpaired Student’s *t*-test (**e**–**h**,**n**,**o**), two-tailed Wilcoxon rank sum test (**j**,**l**) or one-way ANOVA (**q**). Data are presented as the mean ± s.e.m. Source Data

Furthermore, *Ets1* deficiency enhanced OT-I and T_ex_ (but not T_pex_) cell accumulation in the TME but not spleen or tdLN (Fig. 3b,c and Extended Data Fig. 4g–j). Intratumoral *Ets1*-deficient cells also showed increased expression of markers associated with effector function, cytotoxicity and proliferation (Fig. 3d,e and Extended Data Fig. 4k–n), a result that is in agreement with the observed increased percentage of proliferative T_ex_1 cells (Fig. 3a). We next tested the extent to which *Ets1* deficiency affects T_pex_ to T_ex_ cell differentiation using a secondary transfer assay of purified T_pex_ and T_ex_ cells (Extended Data Fig. 5a). Following transfer of *Ets1*-deficient T_pex_ cells, the numbers of total OT-I and T_ex_ cells that developed from T_pex_ cells^5^ increased, a finding associated with more extensive proliferation (Fig. 3f and Extended Data Fig. 5b). Moreover, transfer of *Ets1*-deficient T_ex_ cells resulted in enhanced T_ex_ (and total OT-I) cell accumulation that was accompanied by more proliferation (Fig. 3f and Extended Data Fig. 5c). These analyses suggest that ETS1 is a gatekeeper for T_pex_ to T_ex_ cell differentiation and T_ex_ cell accumulation.

To test therapeutic effects, we performed adoptive cell therapy experiments. Transfer of *Ets1*-deficient OT-I cells or pmel cells (recognizing the B16 melanoma antigen gp100) reduced B16-OVA and B16-F10 tumour growth, respectively (Fig. 3g,h). *Ets1-*deficient CAR T cells targeting human CD19 (hCD19) also showed increased therapeutic effects in hCD19-expressing B16 (B16-hCD19) tumours^12,32^ (Fig. 3i). Beyond these melanoma-related models, *Ets1*-deficient OT-I cells improved therapeutic efficacy against OVA-expressing EL4 lymphoma (E.G7-OVA) and Lewis lung carcinoma (LLC-OVA) tumours (Extended Data Fig. 5d), which was associated with enhanced intratumoral OT-I and T_ex_ cell accumulation (Extended Data Fig. 5e–h). Therefore, targeting *Ets1* improves antitumour effects of CTLs in multiple tumour types.

The combinatorial treatment of OT-I cells deficient for *Ets1* with anti-PD-L1 enhanced antitumour effects compared with control groups in B16-OVA and E.G7-OVA tumours (Fig. 3j and Extended Data Fig. 5i), which suggested that targeting *Ets1* in CD8^+^ T cells enhances the ICB response. Accordingly, *ETS1* expression in CD8^+^ T cells had an inverse correlation with ICB responsiveness in patients with melanoma^33^ (Extended Data Fig. 5j). Furthermore, in scRNA-seq profiling of CTLs from patients with advanced basal cell carcinoma (BCC)^34^, anti-PD-1 treatment induced an activated CD8^+^ T cell population that had lower *ETS1* and higher *IFNG* expression than the exhausted population (Fig. 3k and Extended Data Fig. 5k), with similar effects observed in squamous cell carcinoma (SCC) (Extended Data Fig. 5l). Thus, *ETS1* expression negatively correlates with ICB response, a result consistent with observations in mouse models in which targeting *Ets1* overcomes resistance to ICB.

To explore the mechanistic basis of ETS1-dependent effects, we performed ATAC-seq of T_pex_ and T_ex_ cells. TF motif enrichment and footprinting analyses revealed that *Ets1*-deficient cells had enhanced activity of BATF, a potent regulator of CTL effector function^16,28,32^ (Fig. 3l and Extended Data Fig. 5m,n). Accordingly, BATF expression was increased in T_ex_ cells and total OT-I cells targeted with *Ets1* sgRNA (sg*Ets1*) (Extended Data Fig. 5o). Next, we used secondary genetic interaction screens in vivo to identify functionally relevant ETS1 targets (similar to Extended Data Fig. 3u), focusing on perturbations that reversed the enhanced T_pex_ to T_ex_ cell differentiation and T_ex_ cell accumulation (Supplementary Table 10). Targeting *Batf* in *Ets1*-deficient cells rectified both parameters (Extended Data Fig. 5p). To validate these results, we transferred OT-I cells transduced with sgNTC, sg*Ets1*, sg*Batf* or sg*Ets1* with sg*Batf* OT-I cells into B16-OVA tumour-bearing mice and found that targeting both *Ets1* and *Batf* reversed the increased accumulation of total and T_ex_ cells (Fig. 3m and Extended Data Fig. 5q). The increased percentages of GZMB^+^, IFNγ^+^ and Ki67^+^ *Ets1*-deficient cells were also reversed by *Batf* co-targeting (Fig. 3n). Therefore, the ETS1–BATF axis limits T_ex_ cell accumulation and effector responses.

## RBPJ drives the T_ex_1 to T_ex_2 cell transition

**Fig. 4:**
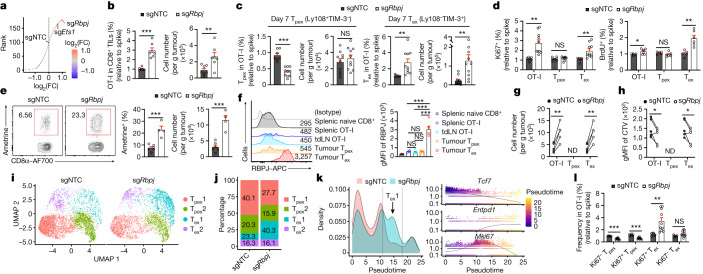
RBPJ drives T_ex_1 to T_ex_2 cell differentiation. **a**, Enrichment (red) or depletion (blue) of sgRNAs in T_ex_1 compared with T_ex_2 cells from scCRISPR screening. **b**,**c**, Relative frequencies and numbers of OT-I cells (*n* = 6 for sgNTC and *n* = 7 for sg*Rbpj*) (**b**) and their T_pex_ and T_ex_ subsets (*n* = 7 for sgNTC and *n* = 10 for sg*Rbpj*) (**c**) in B16-OVA tumours on day 7 (dual-colour transfer system). **d**, Relative frequencies of Ki67^+^ (*n* = 7 for sgNTC and *n* = 10 for sg*Rbpj*; left) and BrdU^+^ (*n* = 5 for sgNTC and *n* = 6 for sg*Rbpj*; right) cells among indicated subsets. **e**, sgNTC or sg*Rbpj* OT-I cells were individually transferred into B16-OVA tumour-bearing mice. Frequency (left) and number (right) of OT-I cells on day 7 after adoptive transfer (*n* = 6 for sgNTC and *n* = 4 for sg*Rbpj*) (single-colour transfer system). **f**, RBPJ expression in OT-I cells from spleen (*n* = 4) or tdLN (n = 5) and T_pex_ or T_ex_ OT-I cells from B16-OVA tumours (*n* = 5) or naive endogenous splenic CD8^+^ T cells (*n* = 4). **g**,**h**, Numbers of total, T_pex_ and T_ex_ OT-I cells (**g**) and CellTrace Violet (CTV) levels (**h**) after T_ex_ cell secondary transfer to B16-OVA tumours (*n* = 5 per group). **i**–**k**, scRNA-seq analysis of sgNTC and sg*Rbpj* OT-I cells from B16-OVA tumours (**i**), cell cluster proportions (**j**) and distribution of sgNTC or sg*Rbpj* OT-I cells and expression dynamics of selected genes across pseudotime (**k**). **l**, Relative frequencies of indicated cell states from B16-OVA tumours (dual-colour transfer system) (*n* = 6 for sgNTC and *n* = 10 for sg*Rbpj*). Data are from representative of three (**b**–**d**,**f**–**h**,**l**) or two (**e**) independent experiments. *P < 0.05, **P < 0.01, ***P < 0.001; two-tailed unpaired Student’s *t*-test (**b**–**e**,**l**), one-way ANOVA (**f**) or two-tailed paired Student’s *t*-test (**g**,**h**). Data are presented as the mean ± s.e.m. Source Data

**Extended Data Fig. 6:**
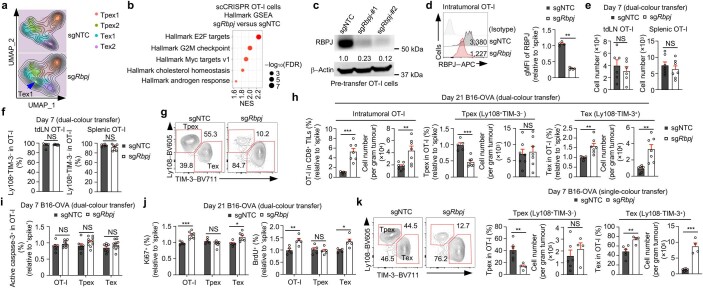
*Rbpj* deficiency selectively promotes Tex cell accumulation. **a**, Contour density plots on UMAP showing distribution of sgNTC and sg*Rbpj* in indicated OT-I cell states as profiled by scCRISPR screening. The arrow indicates the enrichment of the Tex1 cell population in sg*Rbpj* cells. **b**, GSEA enrichment of Hallmark gene signatures in sg*Rbpj* compared with sgNTC OT-I cells (based on gene expression in scCRISPR screening). **c**, Immunoblot analysis of RBPJ expression in OT-I cells transduced with sgNTC or two individual sgRNAs targeting *Rbpj* (sg*Rbpj-*#1 or sg*Rbpj-*#2) cultured for four days. The numbers show abundance of RBPJ (normalized to β-Actin) relative to that of sgNTC OT-I cells. **d**, Relative expression of RBPJ in sgNTC (*n* = 4) or sg*Rbpj* (*n* = 5) intratumoral OT-I cells (dual-colour transfer system). **e**–**g**, Number of OT-I cells in tdLN (*n* = 6 per group) and spleen (*n* = 7 per group) (**e**). Frequencies of Ly108^+^TIM-3^–^ OT-I cells in tdLN and spleen (*n* = 6 for sgNTC and *n* = 7 for sg*Rbpj*). The same sgNTC OT-I cells are presented in Extended Data Fig. 3g (**f**). Flow cytometry analysis of intratumoral Tpex and Tex OT-I cells on day 7 after adoptive transfer (dual-colour transfer system) (**g**). **h**, Relative frequencies and numbers of total intratumoral OT-I cells and their Tpex and Tex cell subsets (dual-colour transfer system) on day 21 after adoptive transfer (*n* = 5 for sgNTC and *n* = 7 for sg*Rbpj*). The same sgNTC OT-I cells are presented in Extended Data Fig. 3d, e. **i**, Relative frequencies of active caspase-3^+^ cells among intratumoral OT-I cells and their Tpex and Tex cell subsets (dual-colour transfer system) on day 7 after adoptive transfer (*n* = 7 for sgNTC and *n* = 10 for sg*Rbpj*). **j**, Relative frequencies of Ki67^+^ (*n* = 5 for sgNTC and *n* = 7 for sg*Rbpj*; left) and BrdU^+^ (*n* = 5 per group; right) cells among indicated intratumoral OT-I cell populations (dual-colour transfer system) on day 21 after adoptive transfer. The same sgNTC OT-I cells are presented in Extended Data Fig. 3q. **k**, Frequencies and numbers of Tpex and Tex OT-I cells after adoptive transfer to B16-OVA tumour-bearing mice (single-colour transfer system) (*n* = 6 for sgNTC and *n* = 4 for sg*Rbpj*). Data are representative of two (**c**,**k**), three (**d**–**g**,**i**) or one (**h**,**j**) independent experiments. NS, not significant; *P < 0.05, **P < 0.01, and ***P < 0.001; two-tailed Kolmogorov–Smirnov test (**b**), two-tailed unpaired Student’s *t*-test (**d**–**f**,**h**–**k**). Data are presented as the mean ± s.e.m. Source Data

Impaired functional and proliferative capacities of T_ex_ cells are a barrier to successful immunotherapy^5–7,9^. We identified *Rbpj* perturbation as a top candidate to increase the T_ex_1 to T_ex_2 cell ratio (Fig. 4a and Supplementary Table 8). *Rbpj* sgRNAs were also enriched in T_ex_1 but not T_ex_2 cells (Extended Data Fig. 6a), which suggested that its targeting may represent a possible mechanism to overcome these immunotherapeutic limitations. Furthermore, *Rbpj*-deficient cells upregulated proliferation signatures (Extended Data Fig. 6b and Supplementary Table 11), which raised the possibility that RBPJ represses intratumoral CTL accumulation. To test this hypothesis, we generated sgRNAs that effectively depleted RBPJ expression (Extended Data Fig. 6c,d and Supplementary Table 7) and observed greater OT-I cell accumulation in the TME but not spleen or tdLN in cells expressing these sgRNAs (Fig. 4b and Extended Data Fig. 6e,f). Furthermore, *Rbpj* deficiency increased T_ex_ cell proportion and accumulation but decreased T_pex_ cell frequency (Fig. 4c and Extended Data Fig. 6g,h). It also increased T_ex_ cell proliferation but did not alter apoptosis (Fig. 4d and Extended Data Fig. 6i,j). Similar effects were observed after transfer of sgNTC or sg*Rbpj* cells separately to tumour-bearing mice (Fig. 4e and Extended Data Fig. 6k). Therefore, RBPJ selectively limits T_ex_ cell accumulation in the TME.

**Extended Data Fig. 7:**
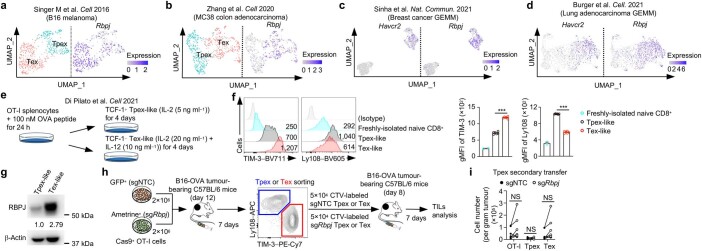
RBPJ is expressed by and mainly functions in Tex cells. **a**, **b**, UMAP plots of CD8^+^ T cells from B16 melanoma (**a**) or MC38 (**b**) tumour. The Tpex and Tex cell subsets and distribution of *Rbpj* expression are indicated. **c**,**d**, UMAP plots showing *Havcr2* and *Rbpj* expression in CD8^+^ T cells from GEMMs of breast cancer (**c**) and lung adenocarcinoma (**d**). **e**–**g**, Schematic for Tpex-like and Tex-like cell generation in vitro (**e**). TIM-3 and Ly108 expression on freshly-isolated naive (CD62L^+^CD44^–^) CD8^+^ T cells (*n* = 2) from spleen or *in vitro*-derived Tpex-like (*n* = 6) and Tex-like (*n* = 6) cells (**f**). Immunoblot analysis of RBPJ protein expression in Tpex-like or Tex-like cells. The numbers show abundance of RBPJ (normalized to β-Actin) relative to that of Tpex-like cells (**g**). **h**,**i**, Diagram of Tpex or Tex cell secondary transfer assays (**h**). Numbers of total intratumoral OT-I cells or their Tpex and Tex cell subsets in the Tpex cell secondary transfer assay (*n* = 7 per group; **i**). Data are representative of three (**f**,**i**) or two (**g**) independent experiments. NS, not significant; ***P < 0.001; two-tailed unpaired Student’s *t*-test (**f**) or two-tailed paired Student’s *t*-test (**i**). Data are presented as the mean ± s.e.m. Source Data

We next examined the regulation of *Rbpj* expression in intratumoral CTLs. *Rbpj* was upregulated in endogenous T_ex_ compared with T_pex_ cells from mouse B16 melanoma^29^ and MC38 colon adenocarcinoma^35^, and was largely co-expressed with *Havcr2* in CD8^+^ T cells from genetically engineered mouse models (GEMMs) of breast cancer^36^ and lung adenocarcinoma^37^ (Extended Data Fig. 7a–d). Furthermore, RBPJ expression in OT-I cells was higher in T_ex_ cells than other intratumoral or peripheral CD8^+^ T cell populations (Fig. 4f). In T_pex_-like and T_ex_-like CD8^+^ T cells generated in vitro^38^ (Extended Data Fig. 7e), concomitant to the expected changes in TIM-3 and Ly108 expression^38^, RBPJ expression was upregulated in T_ex_-like cells (Extended Data Fig. 7f,g), which was consistent with in vivo observations.

In the secondary transfer assay of T_ex_ cells^5,9^ (Extended Data Fig. 7h), *Rbpj* deficiency increased T_ex_ cell accumulation, which was associated with increased proliferation (Fig. 4g,h). Conversely, following transfer of T_pex_ cells, accumulation of T_pex_ and T_ex_ cells remained largely unchanged after targeting *Rbpj* (Extended Data Fig. 7i). Thus, *Rbpj* deficiency results in selective T_ex_ cell accumulation, a finding that provides further support for a cell-intrinsic inhibitory effect of RBPJ on T_ex_ cell accumulation and proliferation.

We next performed scRNA-seq analysis and found a marked increase of T_ex_1 (but not T_ex_2) cells among *Rbpj*-deficient cells (Fig. 4i,j). In pseudotime analysis, *Rbpj*-deficient cells were accumulated in the middle of the differentiation trajectory based on intermediate *Tcf7* and *Entpd1* expression and high *Mki67* expression (Fig. 4k), which was validated by increased Ki67^+^ T_ex_ cell percentage (Fig. 4l). Therefore, *Rbpj* deficiency results in the selective accumulation of T_ex_1 cells.

## Exhaustion increases *RBPJ* expression in human cancers

**Fig. 5:**
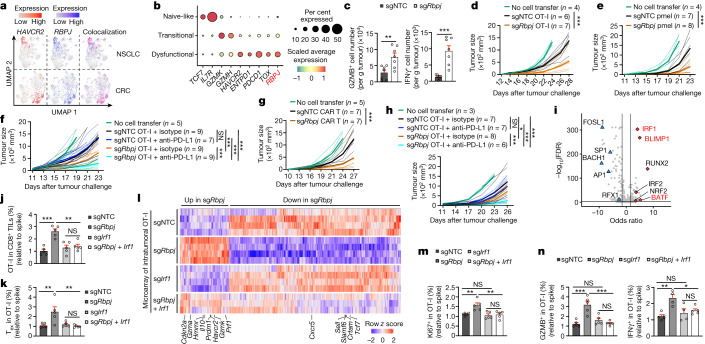
RBPJ deficiency promotes CTL functional reinvigoration. **a**, Expression of *HAVCR2* and *RBPJ* in tumour-derived human CD8^+^ T cells: NSCLC (data from GEO identifier GSE99254) and CRC (data from GEO identifier GSE108989). **b**, Gene expression profiles in melanoma-derived human CD8^+^ T cell populations. **c**, Numbers of GZMB^+^ and IFNγ^+^ sgNTC and sg*Rbpj* OT-I cells isolated on day 7 after adoptive transfer to B16-OVA tumours with OVA/H-2Kb stimulation (*n* = 7 per group; dual-colour transfer). **d**, B16-OVA tumour growth with sgNTC or sg*Rbpj* OT-I cell treatment. **e**, B16-F10 tumour growth with sgNTC (same samples as Fig. 3h) or sg*Rbpj* pmel cell treatment. **f**, B16-OVA tumour growth with indicated treatments. **g**, B16-hCD19 tumour growth with sgNTC (same samples as Fig. 3i) or sg*Rbpj* hCD19-CAR T cell treatment. **h**, E.G7-OVA tumour growth with indicated treatments. **i**, TF motif enrichment analysis (*n* = 3 per group) by ATAC-seq of sg*Rbpj* compared with sgNTC T_ex_ cells. **j**,**k**, Relative frequency of total intratumoral OT-I cells (**j**) or T_ex_ OT-I cells (**k**) transduced with indicated sgRNAs (*n* = 5 per group; dual-colour transfer). **l**, Relative expression of DE genes (sg*Rbpj* compared with sgNTC) in sgNTC (*n* = 4), sg*Rbpj* (*n* = 4), sg*Irf1* (*n* = 4), and sg*Rbpj* with sg*Irf1* (*n* = 3) OT-I cells. **m**,**n**, Relative frequencies of Ki67^+^ (*n* = 5 per group) (**m**) or GZMB^+^ (*n* = 5 per group) and IFNγ^+^ (*n* = 5 for sgNTC, sg*Rbpj* with sg*Irf1*, and *n* = 4 for sg*Rbpj*, sg*Irf1*) OT-I cells (**n**). Data are representative of three (**c**,**d**,**f**,**g**), two (**e**,**j**,**k**,**m**,**n**) or one (**h**) independent experiments. *P < 0.05, **P < 0.01 and ***P < 0.001; two-tailed unpaired Student’s *t*-test (**c**), two-way ANOVA (**d**–**h**) or one-way ANOVA (**j**,**k**,**m**,**n**). Data are presented as the mean ±s.e.m. Source Data

**Extended Data Fig. 8:**
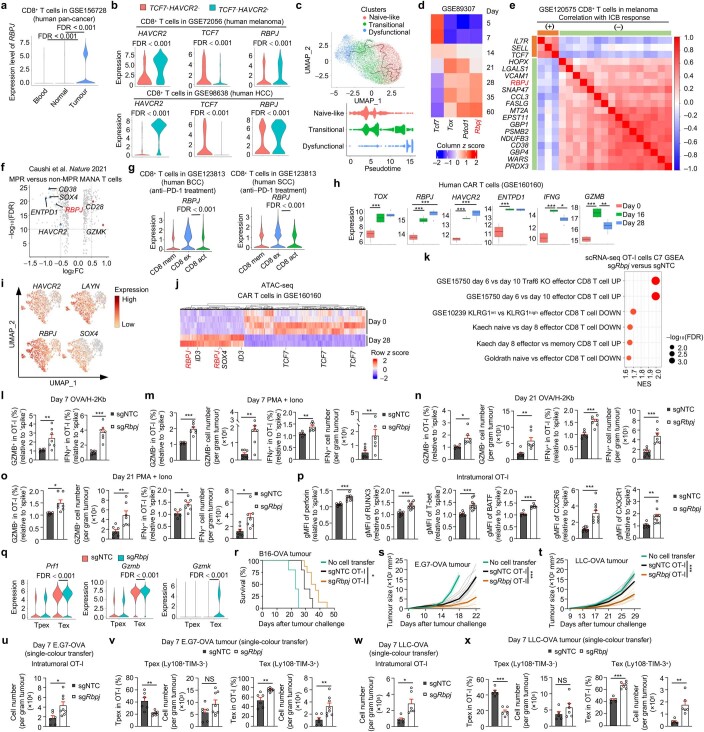
*RBPJ* negatively correlates with immunotherapies in human cancers and suppresses antitumour immunity. **a**, *RBPJ* expression in human CD8^+^ T cells from peripheral blood (blood), tumour-adjacent normal tissues (normal), and tumour tissues. **b**, *HAVCR2*, *TCF7* and *RBPJ* expression in *TCF7*^+^*HAVCR2*^−^ and *TCF7*^−^*HAVCR2*^+^ cells of human CD8^+^ T cells from individuals with melanoma or hepatocellular carcinoma (HCC). **c**, UMAP plot showing cluster analysis (naive-like, transitional and dysfunctional cells) and lineage trajectory of human intratumoral CD8^+^ T cells from individuals with melanoma. The pseudotime of the three clusters along the developmental trajectory is also shown. **d**, Relative expression of *Tcf7*, *Pdcd1*, *Tox* and *Rbpj* in tumour-specific CD8^+^ T cells from a GEMM of liver cancer^13^ at days 5 to 60 after tumour induction. **e**, Correlation matrix displaying the expression of *RBPJ* and genes associated with responsiveness to anti-PD-1 therapy. Kendall rank order correlations are displayed from blue to red. Genes positively (+; orange) or negatively (−; green) associated with response to anti-PD-1 blockade are indicated. **f**, Differentially expressed genes in MANA-specific T cells (derived from patients with NSCLC) with major pathologic response (MPR) compared to those without MPR (non-MPR). **g**, *RBPJ* expression in human CD8^+^ T cell subsets (memory (mem), exhausted (ex) and activated (act)) from patients with BCC or SCC pre- and post-anti-PD-1 treatments. See also Extended Data Fig. 5k. **h**, Expression of *TOX*, *RBPJ*, *HAVCR2*, *ENTPD1*, *IFNG* and *GZMB* from a public bulk RNA-seq dataset of human CAR T cells^46^ at days 0, 16 and 28 after continuous antigen exposure (CAE) in vitro. The boxes stand for 25% to 75% interquartile range (IQR), and the whiskers stand for minimum (25% quantile – 1.5* IQR) to maximum (75% quantile + 1.5* IQR) values, and *n* = 4 biologically independent samples examined over one independent experiment. **i**, UMAP plots showing the expression of *HAVCR2*, *LAYN*, *RBPJ* and *SOX4* in human CAR T cells at day 28 after CAE. **j**. Differentially accessible chromatin regions between days 0 and 28 after CAE, with selective genes showing altered accessibility labeled. **k**, GSEA enrichment of CD8^+^ effector T cell-associated signatures in sg*Rbpj* compared with sgNTC OT-I cells (from scRNA-seq profiling shown in Fig. 4i). **l**, Relative frequencies of GZMB^+^ and IFNγ^+^ intratumoral OT-I cells (dual-colour transfer system, isolated on day 7 after adoptive transfer) after cognate antigen stimulation (*n* = 7 per group). **m**, Relative frequencies and numbers of GZMB^+^ and IFNγ^+^ intratumoral OT-I cells (dual-colour transfer system, isolated on day 7 after adoptive transfer) after PMA + Iono stimulation (*n* = 6 for sgNTC and *n* = 7 for sg*Rbpj*). **n**,**o**, Relative frequencies and numbers of GZMB^+^ and IFNγ^+^ intratumoral OT-I cells (dual-colour transfer system, isolated on day 21 after adoptive transfer) after cognate antigen (**n**; *n* = 5 for sgNTC and *n* = 7 for sg*Rbpj*) or PMA + Iono stimulation (**o**; *n* = 5 for sgNTC and *n* = 7 for sg*Rbpj*). **p**, Relative expression of perforin (*n* = 7 for sgNTC and *n* = 10 for sg*Rbpj*), RUNX3 (*n* = 7 for sgNTC and *n* = 10 for sg*Rbpj*), T-bet (*n* = 7 for sgNTC and *n* = 10 for sg*Rbpj*), BATF (*n* = 6 for sgNTC and *n* = 7 for sg*Rbpj*), CXCR6 (*n* = 7 for sgNTC and *n* = 10 for sg*Rbpj*), and CX3CR1 (*n* = 7 for sgNTC and *n* = 10 for sg*Rbpj*) in total intratumoral OT-I cells (dual-colour transfer system). **q**, Expression of *Prf1*, *Gzmb* and *Gzmk* in Tpex and Tex cell subsets of intratumoral sgNTC and sg*Rbpj* OT-I cells (as profiled by scRNA-seq in Fig. 4i). **r**, Survival analysis of B16-OVA tumour-bearing mice given sgNTC (*n* = 5) or sg*Rbpj* (*n* = 8) OT-I cells. No cell transfer (*n *= 5). **s**, E.G7-OVA tumour growth in mice that received sgNTC (*n* = 8) or sg*Rbpj* (*n* = 8) OT-I cells. No cell transfer (*n* = 4). The same sgNTC OT-I cells are presented in Extended Data Fig. 5d. **t**, LLC-OVA tumour growth in mice given sgNTC (*n *= 9) or sg*Rbpj* (*n* = 10) OT-I cells. No cell transfer (*n* = 4). The same sgNTC OT-I cells are presented in Extended Data Fig. 5d. **u**,**v**, sgNTC (*n* = 6) or sg*Rbpj* (*n* = 8)-transduced OT-I cells were transferred (single-colour transfer system) to E.G7-OVA tumour-bearing mice and analyzed seven days later. Number of total intratumoral OT-I cells (**u**). Frequencies and numbers of Tpex and Tex OT-I cells (**v**). The same sgNTC OT-I cells are presented in Extended Data Fig. 5e, f. **w**,**x**, sgNTC (*n* = 5) or sg*Rbpj* (*n* = 6) OT-I cells were transferred to LLC-OVA tumour-bearing mice (single-colour transfer system) and analyzed seven days later. Number of total intratumoral OT-I cells (**w**). Frequencies and numbers of Tpex and Tex OT-I cells (**x**). The same sgNTC OT-I cells are presented in Extended Data Fig. 5g, h. Data are representative of three (**l**,**m**,**p**,**r**), one (**n**,**o**,**u**–**x**) or two (**s**,**t**) independent experiments. NS, not significant; *P < 0.05; **P < 0.01 and ***P < 0.001; two-tailed Wilcoxon rank sum test (**a**,**b**,**g**,**q**), two-tailed Wald test (**h**), two-tailed Kolmogorov–Smirnov test (**k**), two-tailed unpaired Student’s *t*-test (**l**–**p**,**u**–**x**), log-rank (Mantel-Cox) test (**r**) or two-way ANOVA (**s**,**t**). Data are presented as the mean ± s.e.m. Source Data

We explored whether *RBPJ* expression correlates with exhaustion programmes of human intratumoral T cells. *RBPJ* was increased in CD8^+^ T cells from human tumour tissues^39^, and co-expressed with *HAVCR2* in intratumoral CD8^+^ T cells from patients with non-small cell lung cancer (NSCLC)^40^ and in patients with colorectal cancer (CRC)^41^ (Fig. 5a and Extended Data Fig. 8a). *RBPJ* expression was also upregulated in *TCF7*^−^*HAVCR2*^+^ CTLs from individuals with melanoma^42^ and in patients with hepatocellular carcinoma^43^ (Extended Data Fig. 8b). Intratumoral CD8^+^ T cells from patients with melanoma acquire naive-like, transitional and dysfunctional states^44^, and *RBPJ* expression progressively increased from naive-like to dysfunctional cells (Fig. 5b and Extended Data Fig. 8c). Similarly, in a liver cancer GEMM^13^, *Rbpj* expression was continuously upregulated during T cell exhaustion (Extended Data Fig. 8d). Collectively, these results show that upregulated *RBPJ* expression is a conserved feature of exhausted CD8^+^ T cells in mice and humans.

We next examined correlations between *RBPJ* and genes associated with clinical responses to anti-PD-1 therapy. In melanoma^33^, *RBPJ* clustered with genes negatively associated with responsiveness to anti-PD-1 blockade (Extended Data Fig. 8e). The major pathologic response (MPR) predicts ICB efficacy and is correlated with T cells specific for mutation-associated neoantigens (MANAs)^45^. Accordingly, in NSCLC-derived MANA-specific T cells, *RBPJ* was downregulated in MANA-specific T cells with the MPR (Extended Data Fig. 8f). This result provides further support for the negative correlation between *RBPJ* expression and ICB response. Moreover, individuals with BCC or SCC and treated with anti-PD-1 (ref. ^34^) had lower *RBPJ* expression in the ICB-induced activated T cell population than the exhausted one (Extended Data Fig. 8g). Therefore, low *RBPJ* expression in CD8^+^ T cells is associated with enhanced clinical response to ICB.

We also tested whether *RBPJ* expression correlates with continuous antigen exposure (CAE)-induced CAR T cell exhaustion. Similar to *HAVCR2* and *TOX*, *RBPJ* expression progressively increased and reached the highest levels at day 28, when expression of *IFNG* and *GZMB* reduced (Extended Data Fig. 8h). Furthermore, at day 28 after CAE, *RBPJ* expression largely overlapped with known exhaustion markers^46^ (Extended Data Fig. 8i). Moreover, re-analysis of a public ATAC-seq dataset^46^ revealed that the *RBPJ* gene locus had increased accessibility at day 28 after CAE, similarly to the exhaustion-promoting factors *SOX4* and *ID3* (Extended Data Fig. 8j). These transcriptional and chromatin accessibility analyses revealed that *RBPJ* expression is associated with exhaustion in human CAR T cells, consistent with the negative correlation of *RBPJ* with ICB response.

## *Rbpj* deficiency improves immunotherapy responses

The above analyses suggested that targeting *Rbpj* may enhance CTL effector function and antitumour effects. Accordingly, effector signatures were highly enriched in the absence of *Rbpj* (Extended Data Fig. 8k and Supplementary Table 11). *Rbpj*-deficient cells had increased GZMB^+^ and IFNγ^+^ frequencies and numbers and also upregulated expression of perforin and other effector-associated molecules (Fig. 5c and Extended Data Fig. 8l−p), which indicated enhanced cytotoxic and effector features. scRNA-seq analysis also revealed that *Prf1* (which encodes perforin), *Gzmb* and *Gzmk* were increased in *Rbpj*-deficient T_ex_ cells (Extended Data Fig. 8q). In line with their enhanced effector function, *Rbpj*-deficient OT-I cells better controlled tumour growth and extended the survival of B16-OVA tumour-bearing mice (Fig. 5d and Extended Data Fig. 8r). Similar results were observed after pmel cell transfer to B16-F10 tumour-bearing mice (Fig. 5e). To examine whether targeting *Rbpj* in CTLs enhances ICB response, B16-OVA tumour-bearing mice that received *Rbpj*-deficient OT-I cells were given anti-PD-L1 treatment. This strategy led to enhanced antitumour effects compared with either treatment alone (Fig. 5f). Finally, we tested the effect of *Rbpj* deficiency on the therapeutic efficacy of hCD19 CAR T cells. *Rbpj*-deficient CAR T cells had improved efficacy in limiting tumour growth (Fig. 5g).

To evaluate therapeutic effects in other tumours, we challenged mice bearing E.G7-OVA or LLC-OVA tumours and observed improved antitumour effects from sg*Rbpj* cells (Extended Data Fig. 8s,t). *Rbpj* deficiency also enhanced intratumoral OT-I and T_ex_ (but not T_pex_) cell accumulation in E.G7-OVA and LLC-OVA tumours (Extended Data Fig. 8u–x). Moreover, combinatorial treatment of E.G7-OVA tumour-bearing mice with *Rbpj*-deficient OT-I cells with anti-PD-L1 enhanced antitumour effects compared with control groups (Fig. 5h), which suggested that targeting *Rbpj* in CTLs also boosts ICB response in the lymphoma model. Collectively, these results show that targeting *Rbpj* in CTLs induces potent antitumour effects.

## NOTCH-independent RBPJ signalling

**Extended Data Fig. 9:**
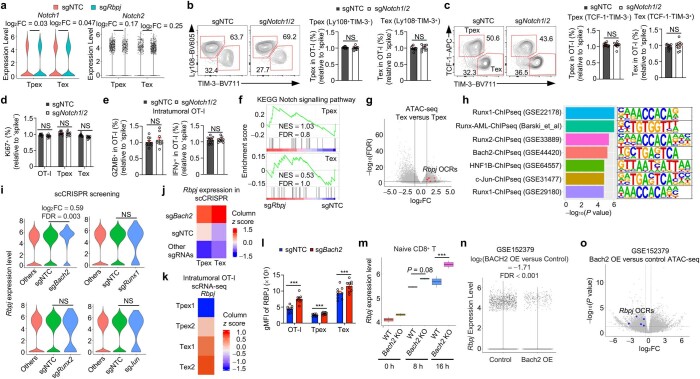
RBPJ acts independently of NOTCH1/2 and is repressed by BACH2. **a**, Expression of *Notch1* and *Notch2* in Tpex and Tex cell subsets among sgNTC or sg*Rbpj* OT-I cells (from scRNA-seq profiling shown in Fig. 4i). **b**–**e**, Relative frequencies of Tpex (Ly108^+^TIM-3^–^ (**b**) or TCF-1^+^TIM-3^–^ (**c**)) and Tex (Ly108^–^TIM-3^+^ (**b**) or TCF-1^–^TIM-3^+^ (**c**)) cells among sgNTC or sg*Notch1/2* intratumoral OT-I cells (dual-colour transfer system). Relative frequencies of Ki67^+^ cells among indicated intratumoral OT-I populations (**d**). Relative frequencies of GZMB^+^ and IFNγ^+^ intratumoral OT-I cells (**e**) (*n* = 10 per group). **f**, GSEA enrichment plots showing no enrichment of KEGG NOTCH signature in sg*Rbpj* compared with sgNTC Tpex cells or sg*Rbpj* compared with sgNTC Tex cells (from scRNA-seq profiling shown in Fig. 4i). **g**, Differential chromatin accessibility profiles of Tex compared with Tpex cells from B16-OVA tumours. Upregulated (red) chromatin accessibility regions of *Rbpj* are labeled. **h**, Enrichment of transcriptional regulators in regions of the *Rbpj* locus (see Methods). **i**, *Rbpj* expression in sg*Bach2*, sg*Runx1*, sg*Runx2* or sg*Jun* OT-I cells compared with OT-I cells transduced with sgNTC and other sgRNAs (sgRNAs for all the other perturbations combined) in scCRISPR screening. **j**, Relative expression of *Rbpj* in Tpex and Tex cell subsets of Cas9-expressing OT-I cells transduced with sg*Bach2*, sgNTC and other sgRNAs (sgRNAs for all the other perturbations combined) from scCRISPR screening. **k**, Relative expression of *Rbpj* in indicated cell states (from scRNA-seq profiling sgNTC cells shown in Fig. 4i). **l**, RBPJ expression in indicated sg*Bach2* (*n* = 9)-transduced intratumoral OT-I cell populations (dual-colour transfer system). **m**, *Rbpj* expression in wild-type (WT) or *Bach2*-deficient (*Bach2* KO) naive CD8^+^ T cells after 0, 8 and 16 h after TCR stimulation. The boxes stand for 25% to 75% interquartile range (IQR), and the whiskers stand for minimum (25% quantile – 1.5* IQR) to maximum (75% quantile + 1.5* IQR) values. **n**, *Rbpj* expression in control and *Bach2* overexpressing (OE) CD8^+^ T cells. **o**, Differential chromatin accessibility in *Bach2* OE CD8^+^ T cells compared with control CD8^+^ T cells. Downregulated (blue) chromatin accessibility regions in the *Rbpj* locus are labeled. Data are representative of two (**b**–**e**) or three (**l**) independent experiments. NS, not significant; two-tailed Wilcoxon sum-rank test (**a**,**i**,**n**), two-tailed unpaired Student’s *t*-test (**b**–**e**,**l**) or two-tailed moderated *t*-test (**m**). Data are presented as the mean ± s.e.m. Source Data

As RBPJ has both NOTCH-dependent and NOTCH-independent functions^47^, we examined *Notch1* and *Notch2* (*Notch1/2*) expression. In contrast to *Rbpj*, *Notch1/2* expression was comparable in T_pex_ and T_ex_ cells (Extended Data Fig. 9a). Additionally, *Notch1/2* co-targeting did not alter the percentages of T_pex_, T_ex_ or Ki67^+^ cells, or cells expressing GZMB or IFNγ (Extended Data Fig. 9b–e). Furthermore, *Rbpj*-deficient T_pex_ and T_ex_ cells had largely unaltered *Notch1/2* expression and NOTCH signalling signature (Extended Data Fig. 9a,f). Therefore, RBPJ functions independently of NOTCH signalling in intratumoral CTL responses.

To identify alternative mechanisms that regulate RBPJ signalling, we performed ATAC-seq analysis. Multiple OCRs in the *Rbpj* locus had increased chromatin accessibility in T_ex_ compared with T_pex_ cells (Extended Data Fig. 9g), which was consistent with the observed increase in *Rbpj* expression in T_ex_ cells (Fig. 4f). TF motif enrichment analysis of these OCRs revealed enrichment for BACH2, RUNX and JUN (Extended Data Fig. 9h), whereas our scCRISPR results showed that targeting *Bach2* (but not *Runx1*, *Runx2* or *Jun*) increased *Rbpj* expression in OT-I, T_pex_ and T_ex_ cells (Extended Data Fig. 9i,j). Furthermore, *Bach2* and *Rbpj* showed reciprocal expression in T_pex_ and T_ex_ cell subsets (Fig. 1j and Extended Data Fig. 9k), which collectively suggested that BACH2 may inhibit RBPJ expression. Indeed, targeting *Bach2* upregulated RBPJ expression in total OT-I, T_pex_ and T_ex_ cells (Extended Data Fig. 9l). Furthermore, *Rbpj* expression was upregulated in TCR-stimulated *Bach2*-deficient CD8^+^ T cells^48^ (Extended Data Fig. 9m). Conversely, *Bach2* overexpression^14^ dampened *Rbpj* expression and gene accessibility (Extended Data Fig. 9n,o). Therefore, BACH2 is necessary and sufficient for inhibiting *Rbpj* expression.

## RBPJ inhibits IRF1 activity

**Extended Data Fig. 10:**
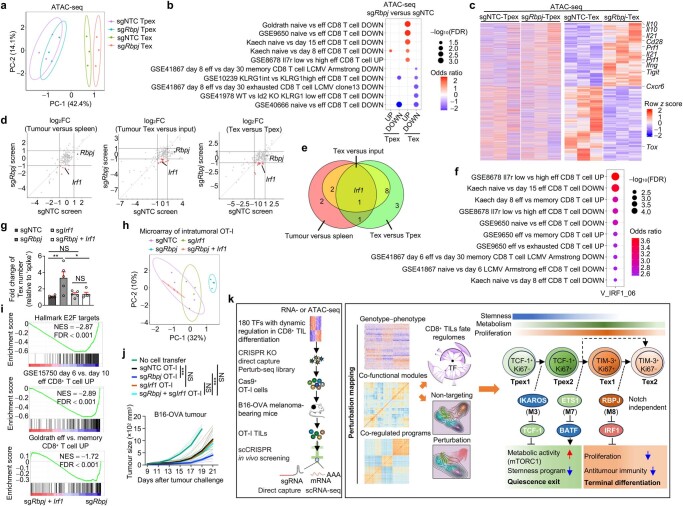
RBPJ–IRF1 axis impinges upon Tex cell accumulation. **a**–**c**, Principal component analysis (PCA) plot showing chromatin alterations in sgNTC (*n* = 3) and sg*Rbpj* (*n* = 3) Tpex and Tex intratumoral OT-I cells (dual-colour transfer system), with the percentage of variance shown (**a**). Peak set enrichment analysis of effector-function-related pathways in Tpex and Tex cell subsets (**b**). Relative intensity of differentially accessible peaks in *Rbpj*-deficient compared with control Tex cells (Tpex peak intensity profiles are shown as reference). Selective genes associated with effector function that display enhanced chromatin accessibility in *Rbpj*-deficient Tex cells are labeled (**c**). **d**, **e**, Sectored scatter plot of gene-level log_2_FC from sgNTC and sg*Rbpj* OT-I cells in genetic interaction screening. The tumour compared with spleen, tumour Tex cells compared with input, and Tex compared with Tpex cell are shown. *Rbpj* and *Irf1* are labeled as described in the text (**d**). Venn diagram showing the number of overlapping candidates from genetic interaction screening with the indicated comparisons (**e**). **f**, OCRs upregulated in sg*Rbpj* compared with sgNTC Tex OT-I cells were analyzed for IRF1 binding motif (V_IRF1_06, from TRANSFAC database), followed by mapping to the nearest genes. Functional enrichment analysis of CD8^+^ T cell effector-function-associated pathways of these genes. **g**, Relative number of Tex cells (dual-colour transfer system) (*n* = 5 per group). **h**, PCA plot showing transcriptome changes in sgNTC (*n* = 4; co-transferred cells from the sg*Irf1* group), sg*Rbpj* (*n* = 4), sg*Irf1* (*n* = 4), and sg*Rbpj* with sg*Irf1* (*n* = 3) intratumoral OT-I cells (dual-colour transfer system), with the percentage of variance shown. **i**, GSEA enrichment plot showing downregulated cell-cycle-associated and effector-function-associated signatures in sg*Rbpj* with sg*Irf1* OT-I cells compared with sg*Rbpj* OT-I cells (from microarray shown in **h**). **j**, B16-OVA tumour growth in mice that received indicated sgRNA-transduced OT-I cells (*n* = 7 per group). No cell transfer group (*n* = 4). **k**, Schematic of in vivo scCRISPR screening and co-functional modules orchestrating heterogeneity and differentiation of intratumoral CTLs. Co-functional modules coordinately regulate gene expression programmes underlying the CTL differentiation trajectory. This trajectory is characterized by a progressive loss of stemness, and an increase in metabolism and proliferative capacity in Tpex2 and Tex1 cells, which are decreased in Tex2 cells. IKAROS (from TF M3) and ETS1 (from TF M7) reciprocally regulate the transition from Tpex to intermediate Tex1 cells, which requires quiescence exit of Tpex cells. Moreover, NOTCH-independent RBPJ (from TF M8)–IRF1 axis mediates Tex1 to Tex2 cell differentiation, associated with reduced proliferation (dotted line marks possible Tpex to Tex2 cell generation that may also arise). Data are representative of two (**g**) or one (**j**) independent experiments. NS, not significant; *P < 0.05, **P < 0.01, and ***P < 0.001; two-tailed Fisher’s exact test (**b**,**f**), one-way ANOVA (**g**) or two-way ANOVA (**j**). Data are presented as the mean ± s.e.m. Source Data

We next determined the downstream mechanisms for RBPJ in CTL differentiation. Peak set enrichment analysis of ATAC-seq profiling data revealed that genes with enhanced chromatin accessibility in *Rbpj*-deficient T_ex_ cells were enriched for pathways related to effector function, whereas fewer changes were noted in T_pex_ cells (Extended Data Fig. 10a,b). Accordingly, effector-function-associated genes had enhanced chromatin accessibility selectively in *Rbpj*-deficient T_ex_ cells (Extended Data Fig. 10c). TF motif analysis of OCRs with increased accessibility in *Rbpj*-deficient T_ex_ cells identified IRF1 as the top enriched motif, along with the effector-function-associated TFs BLIMP1 (ref. ^49^) and BATF^16,28,32^ (Fig. 5i).

Next, we performed a secondary genetic interaction CRISPR screen in vivo (similar to that in Extended Data Fig. 3u), and nominated candidates based on their ability to rectify intratumoral OT-I and T_ex_ cell accumulation and T_ex_ to T_pex_ cell ratio (Extended Data Fig. 10d and Supplementary Table 12). This analysis revealed IRF1 as the only candidate meeting these criteria (Extended Data Fig. 10e). Accordingly, the IRF1-binding motif, identified in OCRs upregulated in sg*Rbpj* T_ex_ cells compared with sgNTC T_ex_ cells in ATAC-seq analysis, was enriched in genes associated with T cell effector function (Extended Data Fig. 10f). Thus, these complementary approaches reveal IRF1 as a top candidate.

To establish the functional relationship between RBPJ and IRF1, we transferred OT-I cells transduced with sgNTC, sg*Rbpj*, sg*Irf1* or sg*Rbpj* with sg*Irf1* into B16-OVA tumour-bearing mice. Targeting both *Rbpj* and *Irf1* reduced the accumulation of total OT-I and T_ex_ cells caused by *Rbpj* deficiency (Fig. 5j,k and Extended Data Fig. 10g). Furthermore, alterations in transcriptome profiles between sgNTC and sg*Rbpj* cells were mitigated by *Irf1* co-targeting (Fig. 5l and Extended Data Fig. 10h), with proliferation-related and effector-function-related pathways also downregulated (Extended Data Fig. 10i). Accordingly, such co-targeting reversed the increased percentages of Ki67^+^, GZMB^+^ and IFNγ^+^ OT-I cells caused by *Rbpj* deficiency in validation experiments (Fig. 5m,n) and the enhanced antitumour effect (Extended Data Fig. 10j). Collectively, these results show that IRF1 is required for *Rbpj* deficiency-induced proliferation and effector function of T_ex_ cells and antitumour effects.

## Discussion

T cell exhaustion represents an adaptive state of hyporesponsiveness that is permissive for persistence in the TME^2^, with terminal differentiation associated with poor antitumour responses. The causal GRN that underlies CTL differentiation and heterogeneity remains elusive. Here we established the functional effects of three transcriptional axes (IKAROS–TCF-1, ETS1–BATF and RBPJ–IRF1) on CTL heterogeneity with important therapeutic implications (Extended Data Fig. 10k). Specifically, IKAROS and ETS1 orchestrate successive steps in the differentiation of T_pex_ cells to proliferative T_ex_1 cells. IKAROS promotes metabolic activation in T_pex_1 cells and their differentiation into T_pex_2 cells, and targeting *Ikzf1* dampened effector function and increased stemness and persistence of intratumoral CTLs, which indicates that *Ikzf1* deficiency probably arrests cells in an excessively quiescent state. Consequently, increased accumulation of *Ikzf1*-deficient cells did not improve antitumour immunity alone or in combination with ICB. Conversely, ETS1 is a gatekeeper for the T_pex_2 to T_ex_1 cell transition, probably by suppressing mTORC1 activity and metabolic reprogramming. Targeting *Ets1* enhanced antitumour effects in multiple immunotherapeutic systems, and *ETS1* expression was negatively associated with ICB response in patients with cancer. Mechanistically, IKAROS and ETS1 limit the respective activities of TCF-1 and BATF. Thus, quiescence exit and metabolic reprogramming represent an underappreciated modality for the transition from stem-like T_pex_ to intermediate T_ex_ cells, and may serve as key therapeutic targets for cancer.

T_ex_ cells are the major intratumoral population and directly contribute to killing tumour cells, but gradually lose proliferative capacity and do not respond to existing immunotherapies^5–7,9^. How to functionally reinvigorate T_ex_ cells to induce antitumour immunity remains unclear. Here we showed that targeting *Rbpj* blocked terminal T_ex_2 cell differentiation but expanded T_ex_1 cells with enhanced proliferation and effector function. *RBPJ* expression correlated with terminal exhaustion in CTLs from patients with cancer and from GEMMs, as well as with hyporesponsiveness to immunotherapies in individuals with cancer. Accordingly, targeting *Rbpj* improved antitumour immunity in multiple therapeutic models. Mechanistically, NOTCH-independent RBPJ signalling acts to suppress IRF1 function. Thus, targeting RBPJ specifically reprogrammes T_ex_ cells and may act in synergy with ICB that targets T_pex_ cells^3–7^.

Together, our study provides a systemic framework of the genetic circuitry and molecular determinants that underlie the functional heterogeneity of intratumoral CTL responses, including three checkpoints for progressive CTL differentiation. Our results highlight the modalities of inducing the quiescence exit of T_pex_ cells and enriching the proliferative T_ex_ cell state for functional reinvigoration of CTL antitumour responses. Of note, the intramodular and intermodular connectivity of co-functional modules may uncover unknown genetic interactions and extend pathway mapping in systems biology, with such approaches being scalable and applicable to other biological systems. Collectively, these results established a perturbation map of progressive differentiation of CD8^+^ T cells in the TME and identified putative actionable targets for the functional reprogramming of T_pex_ and T_ex_ cells to improve cancer immunotherapies.

## Methods

### Mice

The research conducted in this study complied with all of the relevant ethical regulations. The animal protocols were approved by and performed in accordance with the Institutional Animal Care and Use Committee of St. Jude Children’s Research Hospital. C57BL/6, OT-I^50^, pmel^51^ and Rosa26-Cas9 knock-in^52^ mice were purchased from The Jackson Laboratory. Human CD19 CAR-transgenic (CAR-Tg) mice (T cells expressing CARs that consist of anti-hCD19 scFv fragments, the CD8 transmembrane domain and 4-1BB-CD3ζ signalling tail) were provided by T. Geiger^53^. We crossed Rosa26-Cas9 knock-in mice with OT-I, pmel or CAR-Tg mice to generate OT-I-Cas9, pmel-Cas9 and CAR-Tg-Cas9 mice, respectively, that express Cas9 in antigen-specific CD8^+^ T cells. Sex-matched (male or female) mice with predetermined genotypes (not blinded to investigators) were used at 7–12 weeks old unless otherwise noted and assigned randomly to control and experimental groups. All mice were kept in a specific-pathogen-free facility in the Animal Resource Center at St. Jude Children’s Research Hospital. Mice were kept with 12-h light–dark cycles that coincide with daylight in Memphis, TN, USA. The St. Jude Children’s Research Hospital Animal Resource Center housing facility was maintained at 30–70% humidity and 20–25 °C.

### Cell lines

The Plat-E cell line was provided by Y. -C. Liu (La Jolla Institute of Immunology). The B16-OVA cell line was provided by D. Vignali (University of Pittsburgh). The B16-F10 cell line was purchased from the American Type Culture Collection (ATCC). The B16-hCD19 cell line was constructed by transducing B16-F10 cells with an amphotropic virus containing hCD19 and sorting cells with the top 10% of hCD19 expression^12^. The LLC cell line was purchased from the ATCC, and the LLC-OVA cell line was produced by transduction of the parental LLC cell line with the pMIG-II-neo-mOVA containing OVA protein fused with GFP, followed by sorting of GFP-expressing cells^54^. All of the abovementioned cell lines were cultured in Dulbecco’s modified essential medium (DMEM) (Gibco) supplemented with 10% (v/v) FBS and 1% (v/v) penicillin–streptomycin. The E.G7-OVA (derivative of EL4) cell line was purchased from the ATCC and cultured in RPMI 1640 medium (Gibco) supplemented with 10% (v/v) FBS and 1% (v/v) penicillin–streptomycin. No commonly misidentified cell lines were used in this study (International Cell Line Authentication Committee). Cell lines used in this study were not independently authenticated or tested for mycoplasma contamination.

### Flow cytometry

For analysis of surface markers, cells were stained in PBS (Gibco) containing 2% FBS. Surface proteins were stained for 30 min at room temperature. For TF staining, cells were stained for surface molecules, fixed using 2% paraformaldehyde (Thermo Fisher Scientific) for 30 min at room temperature and permeabilized using 90% ice-cold methanol for 30 min on ice. Cells were stained with primary anti-RBPJ (1:100) antibody for 30 min at room temperature followed by staining with goat anti-rabbit IgG (H+L) (1:1,000) for another 30 min at room temperature. For pS6 ex vivo staining, tumour-bearing mice were euthanized and a small portion of tumour was collected and fixed immediately in 2% paraformaldehyde (Thermo Fisher Scientific) for 30 min at room temperature and permeabilized using 90% ice-cold methanol for 30 min on ice. Cells were stained for surface molecules and anti-pS6 (S235/236) (1:100) for 30 min at room temperature. Intracellular staining for cytokines was performed using a BD CytoFix/CytoPerm fixation/permeabilization kit (BD Biosciences) after stimulation with ionomycin (Sigma-Aldrich) and phorbol 12-myristate 13-acetate (PMA; Sigma-Aldrich) in the presence of GolgiSTOP (BD Bioscience) for 4 h or stimulation with OVA/H-2Kb (1 μM) in the presence of GolgiSTOP for 5 h. Active caspase-3 staining was performed using instructions and reagents from an Active Caspase-3 Apoptosis kit (BD Biosciences). BrdU staining (pulsed for 18 h for intratumoral OT-I analyses on day 7 or 21 after adoptive transfer) was performed according to the manufacturer’s instructions using reagents from an APC BrdU Flow kit (BD Biosciences). 7-AAD (A9400, 1:200, Sigma-Aldrich) or fixable viability dye (65-0865-14; 1:1000, eBioscience) was used for dead-cell exclusion. The following antibodies from eBioscience were used: PE–anti-TOX (TXRX10, 12-6502-82, 1:100); APC–anti-perforin (OMAK-D, 17-9392-80, 1:200); PE-cyanine 7–anti-TIM-3 (RMT3-23, 25-5870-82, 1:400); PE–anti-CD244.2 (2B4; 244F4, 12-2441-82, 1:400); eFluor 450–anti-CD71 (R17217(RI7 217.1.4), 48-0711-82, 1:400); PE-cyanine 7–anti-CD44 (IM7, 25-0441-82, 1:400); PerCP-eFluor 710–anti-CD39 (24DMS1, 46-0391-82, 1:400); PerCP-eFluor 710–anti-BATF (MBM7C7, 46-9860-42, 1:100); PE-cyanine 7–anti-T-bet (4B10, 25-5825-82, 1:100); Alexa Fluor 647–goat anti-rabbit IgG (H+L) (A21245, 1:1,000); and Alexa Fluor Plus 405–goat anti-rabbit IgG (H+L) (A48254, 1:1,000). The following antibodies from BioLegend were used: Alexa Fluor 700–anti-CD8α (53-6.7, 100730, 1:400); Brilliant Violet 785–anti-TCRβ (H57-597, 109249, 1:400); Brilliant Violet 650–anti-CD45.1 (A20, 110736, 1:400); APC–anti-TCR-Vα2 (B20.1, 127810, 1:400); APC–anti-Ly108 (330-AJ, 134610, 1:400); Brilliant Violet 711–anti-CD366 (TIM-3) (RMT3-23, 119727, 1:400); Brilliant Violet 421–anti-CX3CR1 (SA011F11, 149023, 1:400); Brilliant Violet 421–anti-CD279 (PD-1) (29 F.1A12, 135217, 1:400); PE–anti-CD62L (MEL-14, 104408, 1:400); PE-cyanine 7–anti-CD98 (4F2, 128214, 1:400); PE–anti-CD186 (CXCR6) (SA051D1, 151104, 1:400); PE–anti-TNF (MP6-XT22, 506306, 1:400); Alexa Fluor 647–anti-GZMB (GB11, 515405, 1:100); PE–anti-IKAROS (2A9/IKAROS, 653304, 1:200); Pacific Blue–anti-Ki67 (16A8, 652422, 1:400); and Brilliant Violet 650–anti-CD11c (N418, 117339. The following antibodies from BD Biosciences were used: Alexa Fluor 647–anti-active caspase-3 (C92-605, 560626, 1:100); Brilliant Violet 605–anti-Ly108 (13G3, 745250, 1:400); and Alexa Fluor 647–anti-BrdU (3D4, 560209, 1:200). VioletFluor 450–anti-IFNγ (XMG1.2, 75-7311-U100, 1:400) was from Tonbo Bioscience, APC–anti-RUNX3/CBFA3 (527327, IC3765A, 1:100) was from R&D Systems, and anti-RBPJ (D10A4, 5313 T), Alexa Fluor 647–anti-TCF-1 (C63D9, 6709, 1:100) and APC–anti-pS6 (S235/236) (D57.2.2E, 14733, 1:100) were from Cell Signaling Technology. To monitor cell division, T_pex_ or T_ex_ cells were labelled with CellTrace Violet (Life Technologies). For mitochondrial staining, TILs were isolated on day 7 after OT-I adoptive transfer and then incubated for 30 min at 37 °C with 10 nM MitoTracker Deep Red (Life Technologies) or 100 nM MitoSOX (Life Technologies) together with staining surface markers. Flow cytometry data were acquired using BD FACSDiva software (v.8) on a LSRII, Symphony A3 or LSR Fortessa (BD Biosciences) and were analysed using FlowJo (v.10.8.1; Tree Star).

### Naive T cell isolation and viral transduction

Naive Cas9-expressing OT-I, pmel or hCD19 CAR-Tg T cells were isolated from the spleen and peripheral lymph nodes of OT-I-Cas9, pmel-Cas9 and CAR-Tg-Cas9 mice using a naive CD8α^+^ T cell isolation kit (Miltenyi Biotec) according to the manufacturer’s instructions. Purified naive OT-I, pmel and hCD19 CAR-Tg T cells were activated in vitro for 18–20 h with 10 μg ml^–1^ anti-CD3 (2C11; Bio-X-Cell), 5 μg ml^–1^ anti-CD28 (37.51; Bio-X-Cell) before viral transduction. Viral transduction was performed by spin-infection at 900*g* at 25 °C for 3 h with 10 μg ml^–1^ polybrene (Sigma-Aldrich). For transduction with two different sgRNAs, the two sgRNA viruses were mixed together and transduced by spin-infection at 900*g* at 25 °C for 3 h with 10 μg ml^–1^ polybrene (Sigma-Aldrich). After transduction, cells were cultured in T cell medium with human IL-2 (20 IU ml^–1^; PeproTech), mouse IL-7 (12.5 ng ml^–1^; PeproTech) and mouse IL-15 (25 ng ml^–1^; PeproTech) for 4 days. Transduced cells were sorted based on the expression of Ametrine, GFP or mCherry (as indicated in the figure legends) using a Reflection cell sorter (iCyt) before adoptive transfer into recipient mice. sgRNAs were designed using an online tool (https://portals.broadinstitute.org/gppx/crispick/public), and the sgRNAs used in this study are listed in Supplementary Table 13. The retroviral sgRNA vector was previously described^16,23^. Retrovirus was produced by co-transfecting Plat-E cells with the core plasmid (sgRNA plasmid or pMIG-overexpressing plasmid) and the helper plasmid pCL-Eco (Addgene, no. 12371) and was collected 72 h after transfection.

### Adoptive T cell transfer

B16-OVA tumour cells (5 × 10^5^) were subcutaneously injected into the right flank of C57BL/6 mice. At day 12 after tumour inoculation, a total of 4 × 10^6^ retrovirus-transduced OT-I cells were adoptively transferred intravenously to the B16-OVA tumour-bearing mice. In the dual-colour transfer system to establish cell-intrinsic effects, OT-I cells transduced with the indicated sgRNAs labelled with Ametrine were mixed at a 1:1 ratio with OT-I cells transduced with sgNTC labelled with GFP (called spike), followed by adoptive transfer to the B16-OVA tumour-bearing mice. TILs were collected for cellular assays (see below) as indicated in the figures and figure legends. To calculate FC values in the dual-colour transfer system, the frequency of indicated population or gMFI of indicated protein is shown relative to spike (sgNTC) cells from the same host. Specifically, the proportion of sgRNA-transduced cells was divided by the proportion of spike cells and further normalized to the ratio of pre-transfer input samples. The quantification of cell number was performed by calculating the numbers of indicated sgRNA-transduced cells and the sgNTC-transduced spike cells from the same host, followed by normalization to the tumour weight^23^. The numbers of sgNTC-transduced cells and spike cells from the same host in control group were comparable and are not depicted in the manuscript. For the single-colour transfer system, the raw percentage and number of indicated population and gMFI of indicated protein are shown. The deletion efficiencies of sg*Rbpj* + *Irf1*, sg*Ikzf1* + *Tcf7* and sg*Ets1* + *Batf* in co-targeting experiments were examined by flow cytometry analyses. In the single-colour transfer system for tumour therapy assays, B16-OVA (5 × 10^5^), B16-F10 (3 × 10^5^) or B16-hCD19 (3 × 10^5^) melanoma cells were subcutaneously injected into the right flank of C57BL/6 mice. On day 12 after tumour inoculation, mice bearing tumours of a similar size were randomly divided into indicated groups (8–10 mice per group). Then, OT-I (for the treatment of B16-OVA melanoma), pmel (for the treatment of B16-F10 melanoma) or hCD19 CAR-Tg (for the treatment of B16-hCD19 melanoma) CD8^+^ T cells (4 × 10^6^) transduced with sgNTC or the indicated sgRNAs (with the same fluorescent reporter protein) were adoptively transferred individually to tumour-bearing mice. For analysis of other tumour models, E.G7-OVA (5 × 10^5^) or LLC-OVA (5 × 10^5^) cells were subcutaneously injected into the right flank of sex-matched C57BL/6 mice. Seven days after tumour inoculation^54,55^, mice bearing tumours of a similar size were randomly divided into indicated groups (8–10 mice per group). Then, OT-I cells (2 × 10^6^ for E.G7-OVA and 4 × 10^6^ for LLC-OVA) transduced with sgNTC or the indicated sgRNAs (with the same fluorescent reporter protein) were adoptively transferred individually to tumour-bearing mice. For anti-PD-L1 treatment, the B16-OVA tumour-bearing mice received OT-I cells on day 12 after tumour inoculation and were then treated with anti-PD-L1 (200 μg; clone 10F.9G2, Bio-X-Cell) or IgG isotype control antibody (200 μg; clone LTF-2, Bio-X-Cell) two times on days 15 and 18 after tumour inoculation. Alternatively, E.G7-OVA tumour-bearing mice received OT-I cells on day 7 after tumour inoculation and were then treated with anti-PD-L1 (200 μg; clone 10F.9G2, Bio-X-Cell) or IgG isotype control antibody (200 μg; clone LTF-2, Bio-X-Cell) two times on days 10 and 13 after tumour inoculation. Mice were monitored for tumour growth and survival; tumours were measured every 2 days with digital calipers and tumour volumes were calculated using the following formula^16^: length × width × [(length × width)^0.5^] × π/6. Tumour size limits were approved to reach a maximum of 3,000 mm^3^ or ≤20% of body weight (whichever was lower) by the Institutional Animal Care and Use Committee at St Jude Children’s Research Hospital. To test the effect of *Ikzf1* deficiency on ICB response, OT-I cells transduced with sg*Ikzf1* (GFP^+^) were mixed at a 1:1 ratio with cells transduced with sgNTC (Ametrine^+^) and co-transferred to B16-OVA tumour-bearing mice on day 12 after tumour inoculation, followed by treatment of anti-PD-L1 or isotype control antibody treatment. sgNTC and sg*Ikzf1* intratumoral OT-I cells from the same recipient mice were analysed for various features on day 7 after adoptive transfer.

### TIL isolation

To isolate TILs on day 7 or 21 after adoptive transfer as indicated in the figure legends, B16-OVA melanoma, EG.7-OVA or LLC-OVA tumours were surgically excised, minced and digested with 0.5 mg ml^–1^ collagenase IV (Worthington) plus 200 IU ml^–1^ DNase I (Sigma-Aldrich) for 1 h at 37 °C. Following the digestions, the tumour tissue was passed through 70-μm filters to remove the undigested part. TILs were then isolated by density-gradient centrifugation over Percoll (Life Technologies).

### Measurement of genome editing efficiency

Pre-transfer OT-I cells or TILs isolated from B16-OVA tumours on day 7 after adoptive transfer were used for analyses of genome editing efficiency. Approximately 1 × 10^5^ cells were centrifuged at 2,000 r.p.m. for 5 min and the cell pellets were lysed. These lysates were used to generate gene-specific amplicons with partial Illumina adapters in the first round of PCR, and then indexed in a second round of PCR, followed by running the sample on a Miseq Sequencer System (Illumina) to generate paired 2 × 250 bp reads. Insertion and deletion mutation analysis was performed using CRIS.py (v.2)^56^.

### scCRISPR screening using the retroviral transcriptional factor library

#### Modified dual-guide direct-capture retroviral sgRNA vector (LMA-DC-EFS) design

To generate LMA-DC-EFS, we replaced the hU6-filler region of the previously described retroviral sgRNA vector (with the use of Ametrine as a selection marker)^16,23^ with the mU6-CR1^CS1^ cassette from the pJR85 (Addgene, no. 140095) vector^11^. To facilitate cloning and library construction, the PGK promoter of the resulting vector was further replaced by the EF1α core promoter from the pCLIP-All-EFS-tRFP vector.

#### Selection of 180 TFs for library design

To select the TFs that are potentially involved in CD8^+^ T cell exhaustion in the tumour context, we performed bioinformatics analyses of DE genes, differential accessibility (DA) of the chromatin state and motif enrichment (ME) for TFs (gene ontology term: 0140110 TF regulatory activity) between early and late exhaustion^13^ and between T_pex_ and T_ex_ cells^5,12,14^ using four published datasets from mouse tumour and chronic infection models (Extended Data Fig. 1c). Specifically, DE and DA analyses were performed using the R package DEseq2 (v.1.32.0)^57^, and |log_2_(FC)| > 0.5 and FDR < 0.05 were used as the cut-off values to define DE genes or DA chromatin regions. FIMO from MEME suite (v.4.11.3)^58^ was used for scanning TF motif (TRANSFAC database release 2019) matches in the nucleosome-free regions, and two-tailed Fisher’s exact test (odds ratio > 1.5 and FDR-corrected *P* value < 0.05) was used to determine whether a motif was significantly enriched in DA chromatin regions. For each dataset, a TF enriched in at least two out of three analyses (DE and DA, DE and ME or DA and ME) was nominated as a putative regulator for exhaustion. TFs were then ranked in descending order by the number of datasets in which they were nominated as putative regulators. The 171 top ranked TFs were selected together with 9 manually curated TFs from literature^26,55,59–63^ to construct the final library targeting 180 TFs (see Supplementary Table 1 for details).

#### Dual-guide direct-capture retroviral library construction

For the curated gene list containing 180 TFs, a total of four gRNA sequences distributed on two individual constructs were designed for each gene. To construct the library, a customized oligonucleotide pool containing 720 oligonucleotides targeting those 180 TFs and 40 NTCs (each oligonucleotide contains two guides targeting the same gene or NTC) (Supplementary Table 2) was ordered from Twist Biosciences. The oligonucleotide design follows the overall structure: 5′-PCR adapter-CCACCTTGTTGG-protospacer A–GTTTCAGAGCAGTCTTCGTTTTCGGGGAAGACAAGAAACATGG-protospacer B–GTTTAAGAGCTAAGC-PCR adapter-3′. The dual-guide library was generated using a two-step cloning strategy as previously described^11^. In brief, the PCR-amplified oligonucleotide pool was digested with BstXI and Bpu1102I (Thermo Fisher) and ligated into a similarly digested LMA-DC-EFS vector. The ligation product was then electroporated into Endura Duos (Lucigen) and amplified, and the resulting intermediate library was assessed for quality using next generation sequencing (NGS). For quality control, sgRNA skewing was measured using the script calc_auc_v1.1.py (ref. ^64^) to monitor how closely sgRNAs are represented in a library, and sgRNA distribution was plotted with the area under the curve < 0.7 to pass quality control. The Python script count_spacers.py^65^ was used as an additional measure for quality control. Next, the CR3^cs1^-hU6 insert from pJR89 (Addgene, no. 140096) was isolated by digestion with BsmBI followed by gel extraction. The intermediate library from above was digested with BbsI and treated with rSAP. Finally, the CR3^cs1^-hU6 insert was ligated into the intermediate library vector, purified by isopropanol purification and electroporated into Endura Duos. Electroporated cells were plated overnight at 32 °C, collected the next day and the plasmid library extracted using endotoxin-free maxiprep kits (Qiagen). The amplified library was then validated by NGS as described above.

#### In vivo screening

The in vivo screening approach was modified from previous studies^16,23^. In brief, retrovirus was produced by co-transfecting the dual-guide, direct-capture retroviral library with pCL-Eco in Plat-E cells. At 48 h after transfection, the supernatant was collected and frozen at −80 °C. Cas9-expressing OT-I cells were transduced to achieve 20–30% transduction efficiency. Transduced cells were sorted based on the expression of Ametrine, and an aliquot of 1 × 10^6^ transduced OT-I cells was saved as input. Transduced OT-I cells (4 × 10^6^) were then transferred intravenously to B16-OVA tumour-bearing C57BL/6 mice at day 12 after tumour inoculation. A total of 60 recipient mice was used in 2 experiments combined. Seven days later, donor-derived total OT-I cells were sorted and pooled for scCRISPR analysis. Sixteen reactions (Chromium Next GEM Single Cell 3′ kit (v.3.1), PN-1000268 and 3′ Feature Barcode kit, PN-1000262; 10x Genomics) in total were used for each reaction (see below).

#### Sequencing library preparation

Sorted OT-I cells were resuspended and diluted in 1× PBS (Thermo Fisher Scientific) containing 0.04% BSA (Amresco) at a concentration of 1 × 10^6^ cells per ml. Both the gene expression library and the CRISPR screening library were prepared using a Chromium Next GEM Single Cell 3′ kit with Feature Barcode technology for CRISPR Screening (v.3.1; 10x Genomics). In brief, the single-cell suspensions were loaded onto the Chromium Controller according to their respective cell counts to generate 10,000 single-cell gel beads in emulsion per sample. Each sample was loaded into four separate channels. The resulting libraries were quantified and quality checked using TapeStation (Agilent). Samples were diluted and loaded onto a NovaSeq (Illumina) to a sequencing depth of 500 million reads per channel for gene expression libraries and 200 million reads per channel for CRISPR screening libraries.

#### Data analysis

Alignments and count aggregation of gene expression and sgRNA reads were completed using Cell Ranger (v.6.0.0)^66^. Gene expression and sgRNA reads were aligned using the cellranger count command with default settings. Gene expression reads were aligned to the mouse genome (mm10 from ENSEMBL GRCm38 loaded from 10x Genomics). sgRNA reads were aligned to our scCRISPR KO library using the pattern GGG(BC)GTTT to capture both sgRNA 1 and 2 on the same vector. The quality control report indicated that an average of 26 sgRNA unique molecular identifiers (UMIs) were detected in each cell. Only droplets with >1 sgRNA UMI were used in further analyses. The filtered feature matrices were imported into Seurat (v.4.0.4)^67,68^ to create assays for a Seurat object containing both gene expression and CRISPR guide capture matrices. A third assay summarizing the total gene-level counts of all four sgRNAs for each target gene was also created, followed by pooling of 16 samples using the merge function. Cells were initially quality filtered based on the percentage of mitochondrial reads <10% (to remove dead cells) and the number of detected RNA features <6,000 and UMI feature <60,000 (removing doublets for gene expression), and 82% cells were detected with at least 1 out of 720 sgRNAs in the library. Because there were two sgRNAs (g1 and g2 or g3 and g4) targeting the same gene on each retroviral construct, the presence of sgRNAs derived from the same vector was detected in the majority (81%) of the cells containing two sgRNAs. Cells detected with sgRNAs targeting two or more genes were then removed to avoid interference from multi-sgRNA-transduced cells. A total of 42,209 OT-I cells passed quality filtering and were used for downstream analysis. A median of 185 cells per target gene (median of 35 sgRNA UMIs per singlet) were recovered, along with 5,371 cells with NTC guides. To evaluate the enrichment or depletion of each perturbation compared with NTC, the relative ratio (log_2_(FC)) of cell number with each perturbation (the four sgRNAs targeting the same gene) compared with sgNTC (on average) was calculated and normalized to account for the different numbers of sgRNAs between gene-specific perturbations and NTC. Eight gene perturbations (sg*Ezh2*, sg*Irf4*, sg*Junb*, sg*Klf2*, sg*Stat5a*, sg*Stat5b*, sg*Yy1* and sg*Zbtb32*) with low cell counts (<48) were removed from the network analysis, as around 50–100 cells are sufficient to accurately identify the perturbation phenotype in scCRISPR experiment for most genes^15^. However, the perturbation effects on the percentages of T_pex_1, T_pex_2, T_ex_1 and T_ex_2 cells were analysed for these eight TFs. For cell clustering, the FindClusters function of the Seurat package was used to identify the clusters in OT-I T cells in an unbiased manner. Cluster-specific genes were identified using the FindAllMarkers function of Seurat. Six clusters (Extended Data Fig. 1f) were annotated based on their distinct signatures, shown in Extended Data Fig. 1g,h.

To determine the molecular determinants for intratumoral CTL developmental trajectory, *Tox*^+^ cells were selected for further graph-based clustering^68^ without including a perturbation-specific cluster (Extended Data Fig. 2a). Clusters were annotated as four cellular states (T_pex_1, T_pex_2, T_ex_1 and T_ex_2) based on *Tcf7*, *Havcr2* and *Mki67* expression. Dot plots showing the relative average expression (after scaled normalization) of marker genes in different clusters were visualized using the DotPlot function in the Seurat R package. Pseudotime trajectory analysis was performed using the Slingshot (v.2.0.0) R package^69^ with default settings. Activity scores of gene signatures (such as the Hallmark mTORC1 signalling gene set^70^) were calculated using the AddModuleScore function of the Seurat package for the four cellular states. To visualize the distribution of cells with a specific perturbation (at the gene level) on the UMAP, contour density plots were generated using the ggplot2 (v.3.3.5) R package. Additionally, the positive and negative regulators in each subset were determined by comparing the abundance of sgRNAs with that in the other three subsets, measured by log_2_(FC). Similarly, the positive and negative regulators between two subsets were determined by comparing the abundance of sgRNAs in these two subsets.

#### Network analysis

Differential gene expression analysis was performed on the TF perturbations with sufficient number of cells (≥48) detected (representing a total of 172 TFs). The FindMarker function of Seurat was used for each perturbation compared with NTC. The log_2_(FC) values were used to indicate the regulatory effect of a perturbation on the targeted genes. To identify the regulatory effect on the regulomes for OT-I cell differentiation, differential expression analysis of each of the six clusters in Extended Data Fig. 1f compared with other clusters was first performed. The top 100 DE genes (ranked by log_2_(FC)) in each of the six clusters were combined as crucial genes for intratumoral OT-I cell differentiation (redundant DE genes between different clusters were removed; 369 genes remained). Then, a gene × perturbation matrix (369 × 172) with log_2_(FC) values was constructed to generate a perturbation map by ascertaining the effect of each genetic perturbation on target gene programmes using the following procedures. First, the co-regulated gene programmes were determined using Pearson-correlation-based hierarchical clustering. Four main gene programmes—effector (programme A), exhaustion (programme B), stemness (programme C) and proliferation (programme D)—were annotated based on their enrichment in the corresponding pathways in Fig. 1d. Second, the co-functional TF modules were determined by Spearman-correlation-based hierarchical clustering. A total of nine co-functional modules were defined. Extended Data Fig. 1o depicts the strength of the connections between all nine TF modules and gene programmes. Specifically, the mean log_2_(FC) value of downstream gene expression alterations (for each of the four gene programmes) induced by the individual TF perturbations (compared with sgNTC) were calculated within each of these modules, followed by measuring the averaged values of all perturbations in that module. The strength of the regulation from TF modules to individual gene programmes was visualized using the ggalluvial R package (v.0.12.3), as indicated by the width of the lines connecting them. The positive and negative regulation effects are shown by red and blue lines, respectively, and the height of each TF module shows the overall strength of that module in regulating gene programmes. Six (M2, M3 and M5–M8) of the nine co-functional modules with the strongest effects (either positive or negative) on each of the four gene programmes (A−D) are further highlighted in Fig. 1e. The connectivity between modules was calculated according to the average number of regulatory effects between modules. For example, the number of edges (regulations) between the individual TFs in two modules was aggregated and normalized by the size (number of components) of the two modules. Third, to uncover specific regulation between individual TFs, especially between the putative central hubs^71^, functionally important central hub TFs were identified based on the number of DE genes (|log_2_FC | > 0.5) affected after perturbation of each TF within that module. Cytoscape software (v.3.7.2) was then used to visualize both intramodular and intermodular connectivity (edges), especially through the central hub TFs (nodes).

### T_pex_ and T_ex_ cell secondary transfer assays

C57BL/6 mice were subcutaneously injected with 3 × 10^5^ B16-OVA melanoma cells on day 0. At day 12 after tumour inoculation, a total of 4 × 10^6^ OT-I cells transduced with sgNTC (labelled with GFP or Ametrine) and sg*Rbpj* (labelled with Ametrine), sg*Ets1* (labelled with GFP) or sg*Ikzf1* (labelled with Ametrine) were mixed at a 1:1 ratio and intravenously injected into the same B16-OVA tumour-bearing mice. sgNTC- and sg*Rbpj*-transduced, sg*Ets1*-transduced or sg*Ikzf1*-transduced T_pex_ (Ly108^+^TIM-3^−^) or T_ex_ (Ly108^−^TIM-3^+^) cells among intratumoral OT-I cells were sorted 7 days after adoptive transfer of OT-I cells. After sorting, sgNTC-transduced and sg*Rbpj*-transduced, sg*Ets1*-transduced or sg*Ikzf1*-transduced T_pex_ or T_ex_ cells were mixed at a 1:1 ratio. The mixed T_pex_ or T_ex_ cells were labelled with 5 μM CellTrace Violet at 37 °C for 15 min and resuspended in PBS. A total of 1 × 10^5^ (5 × 10^4^ sgNTC and 5 × 10^4^ sg*Rbpj*, sg*Ets1* or sg*Ikzf1*) mixed T_pex_ or T_ex_ cells were intravenously transferred to C57BL/6 mice that had been subcutaneously implanted with 5 × 10^5^ B16-OVA cells on day 8 before adoptive transfer. TILs were isolated and analysed 7 days after T_pex_ or T_ex_ cell transfer for analysis.

### In vitro TCF-1^−^ T_ex_-like and TCF-1^+^ T_pex_-like cell cultures

To generate TCF-1^−^ T_ex_-like or TCF-1^+^ T_pex_-like OT-I cells, we adopted an established assay^38^. In brief, splenocytes from Cas9-OT-I transgenic mice were pulsed with 100 nM OVA peptide (Macromolecular Synthesis Core Facility, St Jude Children’s Research Hospital) at 1 × 10^6^ cells ml^–1^ in T cell medium (Click’s medium (IrvineScientific) supplemented with 10% FBS (R&D Systems), 55 mM 2-mercaptoethanol (Sigma-Aldrich) and 1× penicillin–streptomycin–l-glutamine (Gibco)) at 37 °C for 24 h. Then, the cells were cultured at 1 × 10^6^ cells ml^–1^ in T cell medium containing either 20 ng ml^–1^ of mouse IL-2 (mIL-2; PeproTech) and 10 ng ml^–1^ of mIL-12 (PeproTech) or 5 ng ml^–1^ of mIL-2 (PeproTech) to generate TCF-1^−^ T_ex_-like or TCF-1^+^ T_pex_-like cells, respectively. Cells were maintained at the above concentration, and cytokines were replenished daily. Four days later, TCF-1^−^ T_ex_-like or TCF-1^+^ T_pex_-like cells were enriched using lymphocyte isolation medium (LSM) to remove dead cells, followed by flow cytometry and immunoblot analyses.

### Protein isolation and immunoblotting

Cells were lysed in RIPA buffer (Thermo Fisher Scientific), resolved in a 4–12% Criterion XT Bis-Tris protein gel (Bio-Rad) and transferred to a PVDF membrane (Bio-Rad). Membranes were blocked using 5% non-fat milk for 1 h at room temperature and then incubated overnight with anti-RBPJ (D10A4, 1:1,000) (Cell Signaling Technology) or anti-β-actin (AC-74, 1:3,000) (Sigma-Aldrich) antibody at 4 °C. Membranes were washed three times with TBST and then incubated with 1:5,000-diluted HRP-conjugated anti-rabbit IgG or HRP-conjugated anti-mouse IgG (Promega) for 1 h at room temperature. Following another three washes with TBST, the membranes were exposed using enhanced chemiluminescence detection reagents (Thermo Fisher Scientific) and images were captured using an ODYSSEY Fc Analyzer (LI-COR).

### scRNA-seq

C57BL/6 mice were subcutaneously implanted with 3 × 10^5^ B16-OVA melanoma cells on day 0. At day 12 after tumour inoculation, a total of 4 × 10^6^ sgNTC-transduced and sg*Ikzf1-*transduced, sg*Ets1*-transduced or sg*Rbpj*-transduced OT-I cells were mixed at a 1:1 ratio (different fluorescent proteins were used between sgNTC and gene-specific perturbation) and intravenously injected into the same B16-OVA tumour-bearing mice. Intratumoral sgRNA-transduced OT-I cells were sorted from the same host and used in three batches for scRNA-seq analysis: (1) sgNTC-transduced and sg*Ikzf1*-transduced cells; (2) sgNTC-transduced and sg*Ets1*-transduced cells; and (3) sgNTC-transduced and sg*Rbpj*-transduced cells. For longitudinal analysis of OT-I cells, at day 7 after tumour inoculation, a total of 4 × 10^6^ sgNTC (GFP^+^)-transduced OT-I cells were intravenously injected into B16-OVA tumour-bearing mice. After 7, 14 or 21 days, intratumoral sgNTC-transduced OT-I cells were sorted and used for scRNA-seq analysis. After cell counting and centrifugation at 2,000 r.p.m. for 5 min, the supernatant was removed and cells were resuspended and diluted in 1× PBS containing 0.04% BSA at concentration of 1 × 10^6^ cells per ml. Single-cell libraries were prepared using a Chromium Single Cell 3′ Library and Gel Bead kit (v.3.1; 10x Genomics). In brief, the single-cell suspensions were loaded onto the Chromium Controller according to their respective cell counts to generate 9,000 single-cell gel beads in emulsion per sample. Each sample was loaded into a separate channel. The cDNA content of each sample after cDNA amplification of 12 cycles was quantified and quality checked using a High-Sensitivity D5000 chip in a TapeStation (Agilent Technologies) to determine the number of PCR amplification cycles to produce a sufficient library for sequencing. After library quantification and quality checking using a D5000 chip (Agilent Technologies), samples were diluted to 3.5 nM for loading onto a HiSeq 4000 (Illumina) with a 2× 100-bp paired-end kit using the following cycles: 28 cycles read 1, 10 cycles i7 index, 10 cycles i5 index and 90 cycles read 2. An average of 300 million reads per sample was obtained (approximately 20,000 reads per cell).

#### Alignment, barcode assignment and UMI counting

The Cell Ranger Single-Cell software suite (v.6.0.0; 10x Genomics) was implemented to process the raw sequencing data from the Illumina HiSeq run^66^. This pipeline performed demultiplexing, alignment (using the mouse genome mm10 from ENSEMBL GRCm38) and barcode processing to generate gene–cell matrices. The Seurat R package (v.4.0.4) was used for downstream analysis. Specifically, for analysis of sg*Rbpj* effects, data from sgNTC and sg*Rbpj* intratumoral OT-I cell samples (each sample was pooled from two tumour-bearing mice) were used for downstream analysis. For analysis of sg*Ikzf1* and sg*Ets1* effects, two sgNTC and two sg*Ikzf1* or sg*Ets1* OT-I cell samples (each sample from one individual tumour-bearing mouse) were used for downstream analysis. Cells with low UMI counts (potentially dead cells with broken membranes) or high UMI counts (potentially two or more cells in a single droplet) were filtered. Potential dead cells with a high percentage (>10%) of mitochondrial reads were also removed. For the sg*Ikzf1* experiment, a total of 28,945 cells (sgNTC-transduced, 14,044 cells; sg*Ikzf1*-transduced, 14,901 cells) were captured, with an average of 3,012 mRNA molecules (UMIs, median: 11,906; range: 1,026−49,991). For the sg*Ets1* experiment, a total of 20,618 cells (sgNTC-transduced, 10,562 cells; sg*Ets1*-transduced, 10,056 cells) were captured, with an average of 2,955 mRNA molecules (UMIs, median: 12,477; range: 1,703−49,993). For the sg*Rbpj* experiment, a total of 19,516 cells (sgNTC-transduced, 10,918 cells; sg*Rbpj*-transduced, 8,598 cells) were captured, with an average of 2,908 mRNA molecules (UMIs, median: 11,327; range: 1,265−49,887). The expression data were normalized using the NormalizeData function in Seurat with scale.factor = 10^6^. Raw and processed scRNA-seq data have been deposited into the GEO database with the series identifier GSE216800.

#### Data visualization

Underlying cell variations derived in *Tox*^+^ intratumoral CTLs (sgNTC-transduced and sg*Ikzf1*-transduced, sg*Ets1*-transduced and sg*Rbpj*-transduced OT-I cells from TILs) in the single-cell gene expression data were visualized using a two-dimensional projection by UMAP with the Seurat R package (v.4.0.4). sgNTC and sg*Ikzf1*, sg*Ets1* or sg*Rbpj* OT-I cells were further clustered and annotated in an unbiased manner as four cellular states (T_pex_1, T_pex_2, T_ex_1 and T_ex_2) based on *Tcf7*, *Havcr2* and *Mki67* expression. Violin and dot plots that represent the expression levels of selective genes were generated using the VlnPlot and DotPlot function, respectively, in the Seurat R package (v.4.0.4). Pathway scores were calculated using the AddModuleScore function in Seurat (v.4.0.4). Ahmed CXCR5^pos^CD8^+^, Ahmed CXCR5^neg^CD8^+^ T cell signatures were curated from literature^3^ and the GSE41978 KLRG1^hi^CD8^+^ T cell signature^72^ is from MSigDB C7 collection. The stemness signature of CD8^+^ T cells from chronic infection was from the literature^20^, whereas the stemness signature in CD8^+^ T cells from tumours were curated by identifying the significantly upregulated genes (log_2_(FC) > 1 and Benjamini–Hochberg corrected *P* value < 0.05) in TCF-1-GFP^+^ compared with TCF-1-GFP^−^ antigen-specific CD8^+^ T cells (GSE114631)^7^. Pseudotime trajectory analysis was performed using default parameters in the Slingshot R package (v.2.0.0)^69^ on the four intratumoral CTL states.

#### Pre-ranked GSEA and Fisher’s exact test

For scRNA-seq analysis, nonparametric two-tailed Wilcoxon rank-sum test was used to compare the gene expression of cells between two genotypes (sg*Ikzf1* compared with sgNTC, sg*Ets1* compared with sgNTC or sg*Rbpj* compared with sgNTC) and then genes in each comparison were ranked based on their log_2_(FC) values. To identify enriched pathways, pre-ranked GSEA^73^, an analysis of GSEA against a user-supplied, ranked list of genes, was then performed with the MSigDB collection using the fGSEA R package (v.1.18.0) for each comparison. Two-tailed Fisher’s exact test was used to examine whether a MsigDB gene set was significantly (*P* < 0.05) enriched among DE genes after genetic perturbation (significantly increased or decreased (|log_2_(FC)| > 0.5 and FDR < 0.05) genes in sg*Ikzf1*-transduced, sg*Ets1*-transduced or sg*Rbpj*-transduced OT-I cells compared with sgNTC cells).

### Comparison of public and in-house datasets for transcriptome and chromatin accessibility of T_pex_ and T_ex_ cells

To test whether T_pex_ and T_ex_ cells identified in our scCRISPR experiments resemble the established features corresponding to T_pex_ and T_ex_ cells from the same B16-OVA tumour model, FC/FC plot analysis was performed to compare gene expression profiles (based on log_2_(FC) values) in T_ex_ and T_pex_ cells from in-house scCRISPR experiments with those from a public dataset (GSE122713) of antigen-specific CD8^+^ T cells in the literature^5^. The Pearson correlation coefficient was calculated to measure their correlation. The chromatin accessibility (by ATAC-seq) of T_pex_ and T_ex_ cells from our model was further compared with that of CD8^+^ T cells from an acute LCMV infection model (GSE160341) that does not induce T cell exhaustion^23^. OCRs with upregulated accessibility in both T_pex_ and T_ex_ cells compared with CD8^+^ T cells in acute LCMV infection (log_2_(FC) > 1, FDR < 0.05) were visualized using the Heatmap function in the ComplexHeatmap R package (v.2.8.0).

### Public dataset analysis to examine the correlation of *ETS1* or *RBPJ* with CD8^+^ TIL exhaustion or responsiveness to ICB

Multiple public scRNA-seq datasets from the GEO and the European Bioinformatics Institute databases were re-analysed to examine the correlation of *Rbpj* (mouse), *RBPJ* (human) and *ETS1* expression with intratumoral CD8^+^ T cell exhaustion. Seurat (v.4.0.4) R package^67^ was used for preprocessing and visualization, similar to that used for the in-house generated data. For transplanted mouse tumours, *Rbpj* expression in *Pdcd1*^+^*Tcf7*^+^*Havcr2*^−^ and *Pdcd1*^+^*Tcf7*^−^*Havcr2*^+^ cells from both B16 melanoma^29^ (GSE86042) and MC38 tumours^35^ (E-MTAB-8832) was visualized using the FeaturePlot function in Seurat. For scRNA-seq datasets from GEMMs for breast cancer^36^ (GSE161983) and lung carcinoma^37^ (GSE164177), *Rbpj* and *Havcr2* expression in CD8^+^ T cells was visualized using the FeaturePlot function in Seurat. For the RNA-seq dataset from a GEMM for liver cancer (GSE89307)^13^, the relative expression of *Tcf7*, *Pdcd1*, *Tox* and *Rbpj* of tumour-specific CD8^+^ T cells after adoptive transfer was visualized using the Heatmap function in the ComplexHeatmap R package (v.2.8.0). For human tumours, *RBPJ* expression was examined in T_pex_ and T_ex_ cells from multiple tumour datasets, including pan-cancer^39^ (GSE156728), NSCLC^40^ (GSE99254), melanoma^42,44^ (GSE72056 and GSE123139) and HCC^43^ (GSE98638). *ETS1* expression was examined in intratumoral CD8^+^ T cells before ICB (pre-ICB) treatment in patients with melanoma (GSE120575)^33^. In the human melanoma dataset (GSE123139), the naive-like, transitional and dysfunctional CD8^+^ T cell annotations were based on the reported markers in each subset^44^ (*TCF7* and *IL7R* in naive-like; *GZMK* for transitional; *HAVCR2* and *ENTPD1* for dysfunctional). Pseudotime analysis was performed using monocle 3 R package (v1.0.0)^74^ with default settings and naive-like cells as the starting point for trajectory inference.

The correlation of the responsiveness to ICB with *RBPJ* expression in CD8^+^ T cells was assessed in multiple human tumour types. For melanoma^33^ (GSE120575), the Pearson correlation between the gene expression of *RBPJ* and other reported markers for regulating ICB progression was performed in CD8^+^ T cells in patients with melanoma treated with ICB. For other skin cancers, such as BCC^34^ (GSE123813) and SCC^34^ (GSE123813), *RBPJ* and *ETS1* expression was compared using violin plots in the CD8^+^ T cell clusters (originally annotated in the literature) after ICB treatment. For lung cancer^45^, the *RBPJ* gene expression changes in MANA-specific CD8^+^ T cells from patients that responded to ICB (assessed using the MPR, which is associated with overall better patient survival^45^) compared with those that failed to respond to ICB was measured. Finally, the correlation of CAR T cell exhaustion with *RBPJ* expression was assessed in an in vitro exhaustion model^46^ (GSE160160). *RBPJ* expression, together with the exhaustion-associated markers *HAVCR2*, *LAYN* and *SOX4* in CAR T cells after CAE for 28 days, was visualized using Loupe Browser (v.6.0.0; 10x Genomics). In the same dataset, gene expression (bulk RNA-seq) of *RBPJ* was assessed between CAR T cells at days 0, 16 and 28 after CAE, with counts of each gene provided in GSE160160. Differential expression analysis was performed using the R package DEseq2 (v.1.32.0) to calculate the log_2_(FC) and *P* values using day 16 compared with day 0, and day 28 compared with day 16. Gene accessibility of *RBPJ* was also assessed between CAR T cells at day 0 and 28 after CAE by analysing the raw ATAC-seq data in GSE160160.

### ATAC-seq

C57BL/6 mice were subcutaneously implanted with 3 × 10^5^ B16-OVA melanoma cells on day 0. At day 12 after tumour inoculation, a total of 4 × 10^6^ sgNTC (labelled with GFP)-transduced and sg*Ikzf1*-transduced, sg*Ets1*-transduced or sg*Rbpj*-(labelled with Ametrine)-transduced OT-I cells were mixed at a 1:1 ratio and intravenously injected into the same B16-OVA tumour-bearing mice. To prepare the ATAC-seq library, intratumoral sgRNA-transduced OT-I cells or their T_pex_ (Ly108^+^TIM-3^−^) and T_ex_ (Ly108^−^TIM-3^+^) subsets were sorted from the same host for ATAC-seq analysis: sgNTC-transduced and sg*Ikzf1*-transduced, sg*Ets1*-transduced or sg*Rbpj*-transduced cells (*n* = 3 biological replicates per group). Sorted cells were incubated in 50 μl ATAC-seq lysis buffer (10 mM Tris-HCl, pH 7.4, 10 mM NaCl, 3 mM MgCl_2_ and 0.1% IGEPAL CA-630) on ice for 10 min. The resulting nuclei were pelleted at 500*g* for 10 min at 4 °C. The supernatant was carefully removed with a pipette and discarded. The pellet was resuspended in 50 μl transposase reaction mix (25 μl 2× TD buffer, 22.5 μl nuclease-free water and 2.5 μl transposase) and incubated for 30 min at 37 °C to allow tagmentation to occur. The DNA was then cleaned up using a Qiagen MinElute kit. The barcoding reaction of the tagmented DNA was run using a NEBNext HiFi kit based on the manufacturer’s instructions and amplified for five cycles as previously described^16,23^ using the same primers. The optimal cycle numbers were determined from 5 μl (of 50 μl) from the previous reaction mix using KAPA SYBRFast (Kapa Biosystems) and a 20-cycle amplification on an Applied Biosystems 7900HT. The remaining 45 μl of PCR reaction was amplified in the same reaction mix using the optimal cycle number, which is determined from the linear part of the amplification curve.

#### Data analysis

ATAC-seq analysis was performed as previously described^16,23^. In brief, 2× 50-bp paired-end reads obtained from NovaSeq were trimmed for Nextera adaptor by trimmomatic (v.0.36; paired-end mode, with parameter LEADING:10 TRAILING:10 SLIDINGWINDOW:4:18 MINLEN:25) and aligned to mouse genome mm9 downloaded from GenCode release M1 (https://www.gencodegenes.org/mouse/releases.html) by BWA (v.0.7.16, default parameters). Duplicated reads were then marked using Picard (v.2.9.4) and only non-duplicated proper paired reads were kept according to SAMtools (parameter ‘-q 1 -F 1804’ v1.9). After adjustment of Tn5 shift (reads were offset by +4 bp for the sense strand and −5 bp for the antisense strand), reads were separated into nucleosome-free, mononucleosome, dinucleosome and trinucleosome as previously described^75^ by fragment size and generated ‘.bigwig’ files by using the centre 80 bp of fragments and scaled to 30 × 10^6^ nucleosome-free reads. Reasonable nucleosome-free peaks and a pattern of mononucleosome, dinucleosome and trinucleosomes on IGV (v.2.4.13) were observed. All samples had approximately 2 × 10^8^ nucleosome-free reads, indicative of good data quality. Next, peaks were called on nucleosome-free reads using MACS2 (v.2.1.1.20160309, with default parameters with ‘–extsize 200–nomodel’). To ensure reproducibility, nucleosome-free regions for each sample were finalized and retained a peak only if it called with a higher cut-off (MACS2 −q 0.05). Consensus peaks for each group were further generated by keeping peaks that were present in at least 50% of the replicates and discarding the remaining, non-reproducible peaks. The reproducible peaks were further merged between sgNTC-transduced and sg*Ikzf1*-transduced, sg*Ets1*-transduced or sg*Rbpj*-transduced T_pex_ or T_ex_ samples if they overlapped by 100 bp and nucleosome-free reads from each sample were counted using bedtools (v.2.25.0). To identify the DA OCRs, the raw nucleosome-free read was first normalized as counts per million followed by DA analysis by implementation of the negative binomial model in the DESeq2 R package^57^. FDR-corrected *P* values < 0.05, |log_2_(FC)| > 0.5 were used as cut-off values for more-accessible or less-accessible regions in sg*Ikzf1*-transduced, sg*Ets1*-transduced or sg*Rbpj*-transduced T_pex_ and T_ex_ cells compared with their sgNTC-transduced counterparts. Principal component analysis (PCA) was performed using the function prcomp in R. To extract T_pex_-selective and T_ex_-selective OCRs in the ATAC-seq dataset of sgNTC and sg*Ikzf1* cells for heatmap visualization, OCRs from the first principal component (which accounted for the difference between sgNTC T_pex_ and sgNTC T_ex_ cells) were first selected, followed by further selection of T_pex_-selective (log_2_(FC) of T_pex_ compared with T_ex_ > 0.5) and T_ex_-selective (log_2_(FC) of T_pex_ compared with T_ex_ < –0.5) OCRs. The DA OCRs in the ATAC-seq data were assigned for the nearest genes to generate a list of DA genes using HOMER software^76^. FDR-corrected *P* values < 0.05, |log_2_(FC)| > 0.5 were used as cut-off values for more-accessible or less-accessible regions in sg*Rbpj*-transduced T_pex_ and T_ex_ cells compared with their sgNTC-transduced counterparts. Functional peak set enrichment was then performed using MSigDB C7 immunological collection for those DA genes. For motif analysis, 1,000 unchanged regions (log_2_(FC) < 0.05 and FDR-corrected *P* value > 0.5) were selected as control regions for each comparison. FIMO from MEME suite (v.4.11.3, ‘–thresh 1e-4–motif-pseudo 0.0001’)^58^ was used for scanning motifs (TRANSFAC database release 2019, only included Vertebrata and not 3D structure-based) matches in the nucleosome-free regions, and two-tailed Fisher’s exact test was used to determine whether a motif was significantly enriched in DA compared with the control regions. For footprinting analysis of TF binding sites, the RGT HINT (v.0.13.2) application was used to infer TF activity and to plot the results^77^. The *Rbpj* gene locus associated OCRs that increased their accessibility in T_ex_ compared with T_pex_ cells were scanned for TF motifs using HOMER software to determine TFs regulating *Rbpj* expression. In-house generated raw and processed ATAC-seq data have been deposited into the GEO database with the series identifier GSE216800.

### Genetic interaction CRISPR–Cas9 screening using retroviral TF library

#### In vivo screening

The in vivo screening approach was modified based on previous studies^16,23^. In brief, Cas9-expressing OT-I cells were co-transduced with the virus containing the TF library (labelled with Ametrine) in combination with a virus containing sgNTC virus (labelled with mCherry) or sg*Ikzf1*, sg*Ets1* or sg*Rbpj* (labelled with GFP) to achieve 20–30% double transduction efficiency. Transduced cells were sorted based on the co-expression of Ametrine and mCherry or Ametrine and GFP, and an aliquot of 5 × 10^5^ co-transduced OT-I cells were saved as input. A total of 4 × 10^6^ sgNTC (co-labelled with Ametrine and mCherry) and sg*Ikzf1*, sg*Ets1* or sg*Rbpj* (co-labelled with Ametrine and GFP) co-transduced OT-I cells were mixed at a 1:1 ratio and intravenously injected into the same B16-OVA tumour-bearing C57BL/6 mice on day 12 after tumour inoculation. A total of 30 recipients were randomly divided into 3 groups as biological replicates. Seven days after adoptive transfer, total OT-I cells from the spleen and total OT-I cells from TILs or their T_pex_ (Ly108^+^TIM-3^−^) and T_ex_ (Ly108^−^TIM-3^+^) cell subsets (for 100–300× cell coverage per sgRNA) were sorted for further analysis.

#### Sequencing library preparation

Genomic DNA was extracted using DNeasy Blood & Tissue kits (Qiagen). Primary PCR was performed using KOD Hot Start DNA polymerase (Millipore) and the following pair of Nextera NGS primers: Nextera NGS read1-DC-F: TCGTCGGCAGCGTCAGATGTGTATAAGAGACAGCTTGGAGAACCACCTTGTTGG; Nextera NGS read2-DC-R: GTCTCGTGGGCTCGGAGATGTGTATAAGAGACAGAGTTGTAAACGGACTAGCCTTATTTC. Primary PCR products were purified using AMPure XP beads (Beckman). A second PCR was performed by using KOD Hot Start DNA polymerase to add adaptors and indexes to each sample. Second PCR products were purified using AMPure XP beads. Hi-Seq 50-bp single-end sequencing (Illumina) was performed.

#### Data analysis

For bulk secondary genetic interaction CRISPR screens with sgNTC, *sgIkzf1*, sg*Ets1* or sg*Rbpj* OT-I cells, FASTQ read files obtained after sequencing were demultiplexed using Hi-Seq analysis software (Illumina) and processed using mageck (v.0.5.9.4) software^78^. Raw count tables were generated using the mageck count command by matching the guide 1 sequence of the aforementioned dual guide scCRISPR KO library (720 sgRNAs from 180 TF genes and 80 NTC sgRNAs). Read counts for sgRNAs were normalized against median read counts across all samples for each screening.

For each gene or sgRNA in the scCRISPR KO library, the log_2_(FC) for enrichment or depletion was calculated using the mageck test command, with the gene-lfc-method parameter as the mean and control-sgrna parameter using the list of NTC sgRNAs. The log_2_(FC) values of each genetic perturbation from sgNTC and *sgIkzf1*, sg*Ets1* or sg*Rbpj* OT-I cell screens were then compared in a FC/FC plot. Two IKAROS-dependent parameters were used for comparison to identify the candidates: (1) intratumoral T_pex_ cells compared with input cells to uncover factors mediating T_pex_ accumulation; and (2) T_pex_ compared with T_ex_ for factors enhancing the T_pex_/T_ex_ cell ratio. Within each parameter, cut-off values were applied in the FC/FC plot to identify those factors that rectified the above parameters in sg*Ikzf1*-transduced OT-I cells (log_2_(FC_sg*Ikzf1*_) < −1) and less effects in sgNTC-transduced OT-I cells (log_2_(FC_sg*Ikzf1*_) < log_2_(FC_sgNTC_) < 1). Two ETS1-dependent parameters were used for comparison to nominate the candidates: (1) intratumoral T_ex_ cells compared with input cells to uncover factors mediating T_ex_ accumulation; and (2) T_ex_ compared with T_pex_ for factors enhancing the T_ex_/T_pex_ cell ratio. Within each parameter, cut-off values were applied in the FC/FC plot to identify those factors that had rectified above parameters in sg*Ets1*-transduced OT-I cells (log_2_(FC_sg*Ets1*_) < −1) and less effects in sgNTC-transduced OT-I cells (log_2_(FC_sg*Ets1*_) < log_2_(FC_sgNTC_) < 1). Three RBPJ-dependent parameters were used for comparison to nominate the candidates: (1) intratumoral OT-I compared with splenic OT-I cells to identify factors with selective accumulation of intratumoral T cells; (2) intratumoral T_ex_ cells compared with input cells to uncover factors mediating T_ex_ accumulation; and (3) T_ex_ cells compared with T_pex_ cells for factors enhancing the T_ex_/T_pex_ cell ratio. Within each parameter, cut-off values were applied in the FC/FC plot to identify those factors that had rescue effects on the above parameters only in sg*Rbpj*-transduced OT-I cells (log_2_(FC_sg*Rbpj*_) < −1), without significant effects in sgNTC-transduced OT-I cells (−1 < log_2_(FC_sgNTC_) < 1).

### Microarray analysis

Mice were challenged with B16-OVA melanoma cells followed by co-adoptive transfer of sgNTC-transduced cells (GFP^+^mCherry^+^) together with sg*Rbpj*-transduced, sg*Irf1*-transduced or sg*Rbpj* + *Irf1*-transduced OT-I cells (all GFP^+^Ametrine^+^) at day 12 after tumour inoculation. OT-I cells (1 × 10^5^) were sorted from TILs 7 days after adoptive transfer (sgNTC, co-transferred cells from the sg*Irf1* group, *n* = 4; sg*Rbpj*, *n* = 4; sg*Irf1*, *n* = 4; sg*Rbpj* + *Irf1*, *n* = 3; pooled from 3 mice per biological replicate). RNA was isolated using a RNeasy Micro kit (Qiagen 74004) following the manufacturer’s instructions. The RNA concentration was measured using an Agilent 2100 bioanalyzer, followed by microarray analysis with a Clariom S mouse array platform (Thermo Fisher Scientific).

#### Transcriptome profiling

To perform microarray analyses between OT-I cells transduced with sgNTC, sg*Rbpj*, sg*Irf1* or sg*Rbpj* + *Irf1*, the gene expression signals were summarized using the robust multi-array average algorithm (Affymetrix Expression Console v.1.1). Differential expression analysis of genes was performed using the lmFit method implemented in the R package limma (v.3.48.3)^79^. FDR was calculated using the Benjamini–Hochberg method. PCA was performed using the function prcomp in R. The PCA plot was generated using the ggbiplot R package (v.0.55). DE genes in sg*Rbpj* compared with sgNTC OT-I cells were defined by the thresholds of |log_2_(FC) | > 0.5 and FDR < 0.05. The expression of these DE genes in the four genotypes was depicted in a heatmap (ComplexHeatmap R package v.2.8.0). GSEA was performed as previously described^80^ using GSEA software (v.4.2.3) and Hallmark collections from the Molecular Signatures Database (MSigDB v.7.4)^70^ (https://www.broadinstitute.org/gsea/msigdb/).

### Statistical analysis for biological experiments

For biological experiment (non-omics) analyses, data were analysed using Prism 8 software (GraphPad) by two-tailed paired Student’s *t*-test (when comparing with the co-transferred spike cells) or two-tailed unpaired Student’s *t*-test (when comparing with the control group) or one-way ANOVA (when comparing more than two groups). Two-way ANOVA was performed for comparing tumour growth curves when the first mouse reached the experimental end point for euthanasia (that is, when tumours measured 15 mm in the longest dimension). The log-rank (Mantel–Cox) test was performed for comparing mouse survival curves. Two-tailed Wilcoxon rank-sum test was applied for differential expression or activity score analysis of scRNA-seq data. Two-tailed Kolmogorov–Smirnov test was used for GSEA. Two-tailed unpaired Student’s *t*-test was used for TF footprinting analysis of ATAC-seq data. *P* < 0.05 was considered significant, and exact *P* values are provided in the source data that accompany this manuscript. In all bar plots, data are presented as the mean ± s.e.m.

### Reporting summary

Further information on research design is available in the Nature Portfolio Reporting Summary linked to this article.

## Online content

Any methods, additional references, Nature Portfolio reporting summaries, source data, extended data, supplementary information, acknowledgements, peer review information; details of author contributions and competing interests; and statements of data and code availability are available at 10.1038/s41586-023-06733-x.

### Supplementary information

Supplementary Fig. 1 Uncropped immunoblot images with size marker indications.

Reporting Summary

Supplementary Tables This zipped file contains Supplementary Tables 1–13 and a Supplementary Table guide.

### Source data

Source Data Fig. 2

Source Data Fig. 3

Source Data Fig. 4

Source Data Fig. 5

Source Data Extended Data Fig. 1

Source Data Extended Data Fig. 2

Source Data Extended Data Fig. 3

Source Data Extended Data Fig. 4

Source Data Extended Data Fig. 5

Source Data Extended Data Fig. 6

Source Data Extended Data Fig. 7

Source Data Extended Data Fig. 8

Source Data Extended Data Fig. 9

Source Data Extended Data Fig. 10

## Data Availability

The data supporting the findings of this study are available within the manuscript and its Supplementary Information. All microarray, scCRISPR screening, ATAC-seq and scRNA-seq data described in the manuscript have been deposited in the NCBI GEO database and are accessible through the GEO SuperSeries access number GSE216800. Public scRNA-seq datasets are available through GSE156728, GSE99254, GSE108989, GSE122713, GSE123813, GSE120575, GSE86042, GSE161983, GSE164177, GSE72056, GSE123139, GSE98638 and E-MTAB-8832. Public bulk RNA-seq datasets are available through GSE160160 and GSE89307. Public ATAC-seq datasets are available through GSE160341. KEGG, C7 immunological, gene ontology and HALLMARK collections were from the mSigDB (https://www.broadinstitute.org/gsea/msigdb/). Source data are provided with this paper.

## Code availability

All code generated for analysis is available from the author upon request.
