# Supplementary material for: Single-cell CRISPR screens in vivo map T cell fate regulomes in cancer
Source: Nature. 2023 Nov 15;624(7990):154–63. doi: 10.1038/s41586-023-06733-x (PMC10700132; doi:10.1038/s41586-023-06733-x)

nature portfolio

https://doi.org/10.1038/s41586-023-06733-x

Supplementary information

Single-cell CRISPR screens in vivo map T cell fate regulomes in cancer

In the format provided by the
authors and unedited

Supplementary Figure 1. Uncropped immunoblot images with size marker indications.

Extended Data Fig. 6c

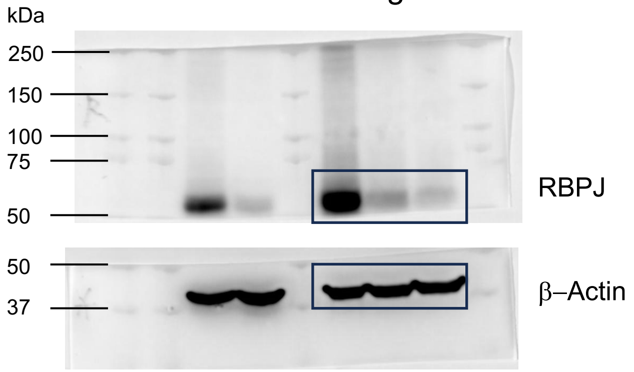

Extended Data Fig. 7g

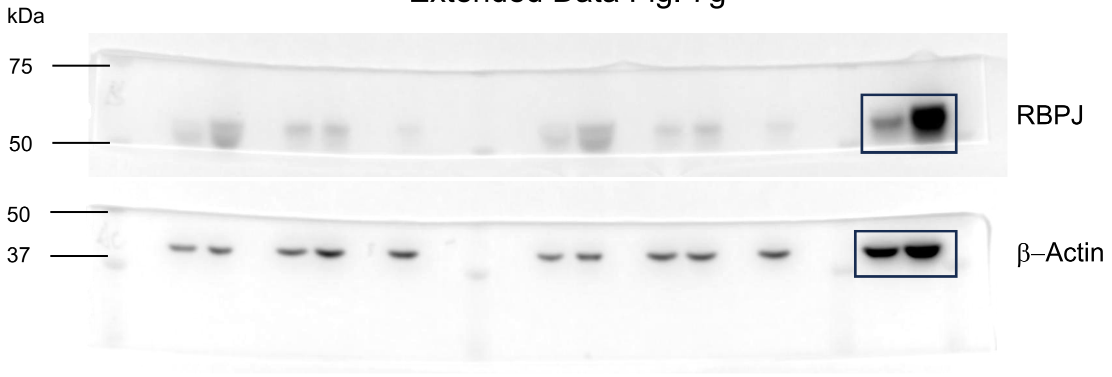

Supplement: Supplementary file 1 — Uncropped immunoblot images with size marker indications. [file 41586_2023_6733_MOESM1_ESM.pdf]
