# Supplementary material for: Single-cell CRISPR screens in vivo map T cell fate regulomes in cancer
Source: Nature. 2023 Nov 15;624(7990):154–63. doi: 10.1038/s41586-023-06733-x (PMC10700132; doi:10.1038/s41586-023-06733-x)
Supplement: Supplementary file 2 — Reporting Summary [file 41586_2023_6733_MOESM2_ESM.pdf]

nature portfolio

Corresponding author(s): Hongbo Chi

Last updated by author(s): Sep 20, 2023

Reporting Summary

Nature Portfolio wishes to improve the reproducibility of the work that we publish. This form provides structure for consistency and transparency in reporting. For further information on Nature Portfolio policies, see our Editorial Policies and the Editorial Policy Checklist.

Statistics

For all statistical analyses, confirm that the following items are present in the figure legend, table legend, main text, or Methods section.

n/a	Confirmed	
☐	☒	The exact sample size (*n*) for each experimental group/condition, given as a discrete number and unit of measurement
☐	☒	A statement on whether measurements were taken from distinct samples or whether the same sample was measured repeatedly
☐	☒	The statistical test(s) used AND whether they are one- or two-sided<br>*Only common tests should be described solely by name; describe more complex techniques in the Methods section.*
☐	☒	A description of all covariates tested
☐	☒	A description of any assumptions or corrections, such as tests of normality and adjustment for multiple comparisons
☐	☒	A full description of the statistical parameters including central tendency (e.g. means) or other basic estimates (e.g. regression coefficient) AND variation (e.g. standard deviation) or associated estimates of uncertainty (e.g. confidence intervals)
☐	☒	For null hypothesis testing, the test statistic (e.g. *F*, *t*, *r*) with confidence intervals, effect sizes, degrees of freedom and *P* value noted<br>*Give P values as exact values whenever suitable.*
☒	☐	For Bayesian analysis, information on the choice of priors and Markov chain Monte Carlo settings
☒	☐	For hierarchical and complex designs, identification of the appropriate level for tests and full reporting of outcomes
☐	☒	Estimates of effect sizes (e.g. Cohen's *d*, Pearson's *r*), indicating how they were calculated

Our web collection on statistics for biologists contains articles on many of the points above.

Software and code

Policy information about availability of computer code

Data collection	BD FACSDIva software (v8) was used to collect flow cytometry data on LSRII, Symphony A3 or Fortessa cytometers (BD Biosciences).
Data analysis	FlowJo v10 (TreeStar) for FACS results;<br>GraphPad Prism v8 for statistics;<br>Affymetrix Expression console v1.1, limma R package v3.48.3 for microarray;<br>DESeq2 R package v. 1.32.0 for RNA-seq and ATAC-seq;<br>Picard (v.2.9.4), BWA (V0.7.16), SAMtools (v1.9),MACS2 V2.1.120160309, bedtools v2.25.0, RGT HINT v0.13.2, MEME v4.11.3, IGV v.2.4.13 for ATAC-seq;<br>Cell Ranger v6.0.0, Loupe Browser v6.0.0, Seurat R package v4.0.4, Slingshot R package v2.0.0, monocle 3 R package v 1.0.0, fGSEA R package v1.18.0, GSEA software v4.2.3, MSigDB v7.4, Cytoscape v.3.7.2, ggalluvial R package v.0.12.3, ggplot2 R package v.3.3.5, ggbiplot R package v0.55 for scRNA-seq and scCRISPR screening;<br>mageck v0.5.9.4 software for bulk CRISPR screening;<br>ComplexHeatmap v2.8.0 for microarray, RNA-seq, ATAC-seq and scCRISPR screening;<br>CRIS.py v2 (https://github.com/patrickc01/CRIS.py) for insertion and deletion (indel) mutation analysis.<br>All codes used for analysis are available from the authors upon request (see Code Availability Statement).

For manuscripts utilizing custom algorithms or software that are central to the research but not yet described in published literature, software must be made available to editors and reviewers. We strongly encourage code deposition in a community repository (e.g. GitHub). See the Nature Portfolio guidelines for submitting code & software for further information.

Data

Policy information about availability of data

All manuscripts must include a data availability statement. This statement should provide the following information, where applicable:

- Accession codes, unique identifiers, or web links for publicly available datasets
- A description of any restrictions on data availability
- For clinical datasets or third party data, please ensure that the statement adheres to our policy

The authors declare that the data supporting the findings of this study are available within the manuscript and its Supplementary Information. All microarray, scCRISPR screening, ATAC-seq and scRNA-seq data described in the manuscript have been deposited in the NCBI Gene Expression Omnibus (GEO) database and are accessible through the GEO SuperSeries access number GSE216800 (https://www.ncbi.nlm.nih.gov/geo/query/acc.cgi?acc=GSE216800).

Public scRNA-seq datasets are available through GSE156728 (https://www.ncbi.nlm.nih.gov/geo/query/acc.cgi?acc=GSE156728), GSE99254 (https://www.ncbi.nlm.nih.gov/geo/query/acc.cgi?acc=GSE99254), GSE108989 (https://www.ncbi.nlm.nih.gov/geo/query/acc.cgi?acc=GSE108989), GSE122713 (https://www.ncbi.nlm.nih.gov/geo/query/acc.cgi?acc=GSE122713), GSE123813 (https://www.ncbi.nlm.nih.gov/geo/query/acc.cgi?acc=GSE123813), GSE120575 (https://www.ncbi.nlm.nih.gov/geo/query/acc.cgi?acc=GSE120575), GSE86042 (https://www.ncbi.nlm.nih.gov/geo/query/acc.cgi?acc=GSE86042), GSE161983 (https://www.ncbi.nlm.nih.gov/geo/query/acc.cgi?acc=GSE161983), GSE164177 (https://www.ncbi.nlm.nih.gov/geo/query/acc.cgi?acc=GSE164177), GSE72056 (https://www.ncbi.nlm.nih.gov/geo/query/acc.cgi?acc=GSE72056), GSE123139 (https://www.ncbi.nlm.nih.gov/geo/query/acc.cgi?acc=GSE123139), GSE98638 (https://www.ncbi.nlm.nih.gov/geo/query/acc.cgi?acc=GSE98638), E-MTAB-8832 (https://www.ebi.ac.uk/biostudies/arrayexpress/studies/E-MTAB-8832). Public bulk RNA-seq datasets are available through GSE160160 (https://www.ncbi.nlm.nih.gov/geo/query/acc.cgi?acc=GSE160160), GSE89307 (https://www.ncbi.nlm.nih.gov/geo/query/acc.cgi?acc=GSE89307). Public ATAC-seq datasets are available through GSE160341 (https://www.ncbi.nlm.nih.gov/geo/query/acc.cgi?acc=GSE160341). KEGG, C7 immunological, GO and HALLMARK collections were from the Molecular Signatures Database (mSigDB) (https://www.broadinstitute.org/gsea/msigdb/).

Human research participants

Policy information about studies involving human research participants and Sex and Gender in Research.

Reporting on sex and gender	N/A
Population characteristics	N/A
Recruitment	N/A
Ethics oversight	N/A

Note that full information on the approval of the study protocol must also be provided in the manuscript.

Field-specific reporting

Please select the one below that is the best fit for your research. If you are not sure, read the appropriate sections before making your selection.

☒ Life sciences ☐ Behavioural & social sciences ☐ Ecological, evolutionary & environmental sciences

For a reference copy of the document with all sections, see nature.com/documents/nr-reporting-summary-flat.pdf

Life sciences study design

All studies must disclose on these points even when the disclosure is negative.

Sample size	Sample sizes were selected based on those used in previous publications (Wei et al. Nature 2019; Huang et al. Cell 2021).
Data exclusions	No data were excluded.
Replication	All the experimental findings were reliably reproduced as validated by at least three biological replicates in at least two independent experiments unless otherwise noted.
Randomization	Age- and sex-matched mice, including samples other than those involving mice, were assigned randomly to experimental and control groups.
Blinding	The investigators were not blinded to group allocation during data collection or analysis. This approach is considered standard for experiments of the type performed in this study, as the genetic background of the input cells must be predetermined prior to analysis.

Reporting for specific materials, systems and methods

We require information from authors about some types of materials, experimental systems and methods used in many studies. Here, indicate whether each material, system or method listed is relevant to your study. If you are not sure if a list item applies to your research, read the appropriate section before selecting a response.

Materials & experimental systems

n/a	Involved in the study
☐	☒ Antibodies
☐	☒ Eukaryotic cell lines
☒	☐ Palaeontology and archaeology
☐	☒ Animals and other organisms
☒	☐ Clinical data
☒	☐ Dual use research of concern

Methods

n/a	Involved in the study
☒	☐ ChIP-seq
☐	☒ Flow cytometry
☒	☐ MRI-based neuroimaging

Antibodies

Antibodies used

1. The following antibodies were used for cell culture: anti-CD3 (2C11; Bio-X-Cell, BE0001-1) and anti-CD28 (37.51; Bio-X-Cell, BE0015-1).

2. The following antibodies were used for in vivo treatments: anti-PD-L1 antibody (10F.9G2, Bio-X-Cell) and rat IgG2b isotype control (LTF-2, Bio-X-Cell).

3. For flow cytometry analysis: 7-AAD (A9400, 1:200, Sigma) or fixable viability dye (65-0865-14; 1:1,000, eBioscience) was used for dead-cell exclusion. The following fluorescent conjugate-labeled antibodies were used: Alexa Fluor 700–anti-CD8α (53-6.7, 100730, 1:400), Brilliant Violet 785–anti-TCRβ (H57-597, 109249, 1:400), Brilliant Violet 650–anti-CD45.1 (A20, 110736, 1:400), PE–anti-CD62L (MEL-14, 104408, 1:400), PE-Cyanine7–anti-CD98 (4F2, 128214, 1:400), Brilliant Violet 421–anti-CX3CR1 (SA011F11, 149023, 1:400), APC–anti-TCR-Vα2 (B20.1, 127810, 1:400), APC–anti-Ly108 (330-AJ, 134610, 1:400), Brilliant Violet 711–anti-TIM-3 (RMT3-23, 119727, 1:400), PE–anti-CD186 (CXCR6) (SA051D1, 151104, 1:400), Brilliant Violet 421–anti-CD279 (PD-1) (29F.1A12, 135217, 1:400), PE–anti-TNF (MP6-XT22, 506306, 1:400), Alexa Fluor 647–anti-granzyme B (GB11, 515405, 1:100), Pacific Blue–anti-Ki67 (16A8, 652422, 1:400), PE–anti-IKAROS (2A9/IKAROS, 653304, 1:200), Brilliant Violet 650–anti-CD11c (N418, 117339, 1:400) (all from Biolegend); PE-Cyanine7–anti-CD44 (IM7, 25-0441-82, 1:400), eFluor 450–anti-CD71 (R17217, 48-0711-82, 1:400), PE-Cyanine7–anti-TIM-3 (RMT3-23, 25-5870-82, 1:400), PE–anti-CD244.2 (2B4; 244F4, 12-2441-82, 1:400), PerCP-eFluor 710–anti-CD39 (24DMS1, 46-0391-82, 1:400), APC–anti-perforin (OMAK-D, 17-9392-80, 1:200), PerCP-eFluor 710–anti-BATF (MBM7C7, 46-9860-42, 1:100), PE-Cyanine7–anti-T-bet (4B10, 25-5825-82, 1:100), PE–anti-TOX (TXRX10, 12-6502-82, 1:100), Alexa Fluor 647–goat anti-rabbit IgG (H+L) (A21245, 1:1,000), Alexa Fluor Plus 405–goat anti-rabbit IgG (H+L) (A48254, 1:1000) (all from eBioscience); Brilliant Violet 605–anti-Ly108 (13G3, 745250, 1:400), Alexa Fluor 647–anti-active caspase-3 (C92-605, 560626, 1:100), Alexa Fluor 647–anti-BrdU (3D4, 560209, 1:200) (all from BD Biosciences); VioletFluor 450–anti-IFNγ (XMG1.2, 75-7311-U100, 1:400) (from Tonbo Bioscience); APC–anti-RUNX3/CBFA3 (527327, IC3765A, 1:100) (from R&D systems); Alexa Fluor 647–anti-TCF1 (C63D9, 6709, 1:100), APC–anti-pS6 (S235/236) (D57.2.2E, 14733, 1:100) (all from Cell Signaling Technology).

4. The following antibodies were used for immunoblot analysis: anti-β-Actin (AC-74, 1:3,000, Sigma-Aldrich) and anti-RBPJ (D10A4, 1:1,000, Cell Signaling Technology). Primary antibodies were detected using HRP-conjugated anti-mouse IgG (W4021, 1:5,000, Promega) or anti-Rabbit IgG (W4011, 1:5,000, Promega).

Validation

1. The following antibodies for cell culture have been validated for the specificity and application by the manufacturers (see detailed reference on the website)
anti-mouse CD3: www.bioxcell.com/invivomab-anti-mouse-cd3e-be0001-1
anti-CD28: www.bioxcell.com/invivomab-anti-mouse-cd28-be0015-1

2. The following antibodies for in vivo treatment have been validated for the specificity and application by the manufacturers (see detailed reference on the website)
anti-mouse PD-L1 (B7-H1): www.bioxcell.com/invivomab-anti-mouse-pd-l1-b7-h1-be0101
rat IgG2b isotype control: www.bioxcell.com/invivomab-rat-igg2b-isotype-control-anti-keyhole-limpet-hemocyanin-be0090

3. The following antibodies for flow cytometry have been validated for the specificity and application by the manufacturers (see detailed reference on the website)
7-AAD: www.sigmaaldrich.com/US/en/product/sigma/a9400
Fixable viability dye: www.thermofisher.com/order/catalog/product/65-0865-14?SID=srch-srp-65-0865-14
Alexa Fluor 700–anti-CD8α: www.biolegend.com/fr-ch/products/alexa-fluor-700-anti-mouse-cd8a-antibody-3387
Brilliant Violet 785–anti-TCRβ: www.biolegend.com/fr-ch/products/brilliant-violet-785-anti-mouse-tcr-b-chain-antibody-17614
Brilliant Violet 650–anti-CD45.1: www.biolegend.com/fr-ch/products/brilliant-violet-650-anti-mouse-cd45-1-antibody-7644
PE–anti-CD62L: www.biolegend.com/fr-ch/products/pe-anti-mouse-cd62l-antibody-386
PE-Cyanine7–anti-CD98: www.biolegend.com/fr-ch/products/pe-cyanine7-anti-mouse-cd98-4f2-antibody-16518
Brilliant Violet 421–anti-CX3CR1: www.biolegend.com/fr-ch/products/brilliant-violet-421-anti-mouse-cx3cr1-antibody-11852
APC–anti-TCR-Vα2: www.biolegend.com/fr-ch/products/apc-anti-mouse-tcr-valpha2-antibody-4851
APC–anti-Ly108: www.biolegend.com/fr-ch/products/apc-anti-mouse-ly108-antibody-15660
Brilliant Violet 711–anti-TIM-3: www.biolegend.com/fr-ch/products/brilliant-violet-711-anti-mouse-cd366-tim-3-antibody-14918
PE–anti-CD186 (CXCR6): www.biolegend.com/fr-ch/products/pe-anti-mouse-cd186-cxcr6-antibody-12545
Brilliant Violet 421–anti-CD279 (PD-1): www.biolegend.com/fr-ch/products/brilliant-violet-421-anti-mouse-cd279-pd-1-antibody-7330
PE–anti-TNF: www.biolegend.com/fr-ch/products/pe-anti-mouse-tnf-alpha-antibody-978
Alexa Fluor 647–anti-granzyme B: www.biolegend.com/fr-ch/products/alexa-fluor-647-anti-human-mouse-granzyme-b-

antibody-6067
Pacific Blue–anti-Ki67: www.biolegend.com/fr-ch/products/pacific-blue-anti-mouse-ki-67-antibody-10553
PE–anti-IKAROS: www.biolegend.com/fr-ch/products/pe-anti-mouse-ikaros-antibody-8308
Brilliant Violet 650–anti-CD11c: www.biolegend.com/en-us/products/brilliant-violet-650-anti-mouse-cd11c-antibody-8840
PE-Cyanine7–anti-CD44: www.thermofisher.com/antibody/product/CD44-Antibody-clone-IM7-Monoclonal/25-0441-82
eFluor 450–anti-CD71: www.thermofisher.com/antibody/product/CD71-Transferrin-Receptor-Antibody-clone-R17217-RI7-217-1-4-Monoclonal/48-0711-82
PE-Cyanine7–anti-TIM-3: www.thermofisher.com/antibody/product/CD366-TIM3-Antibody-clone-RMT3-23-Monoclonal/25-5870-82
PE–anti-CD244.2: www.thermofisher.com/antibody/product/CD244-2-2B4-Antibody-clone-eBio244F4-Monoclonal/12-2441-82
PerCP-eFluor 710–anti-CD39: www.thermofisher.com/antibody/product/CD39-Antibody-clone-24DMS1-Monoclonal/46-0391-82
APC–anti-perforin: www.thermofisher.com/antibody/product/Perforin-Antibody-clone-eBioOMAK-D-Monoclonal/17-9392-80
PerCP-eFluor 710–anti-BATF: www.thermofisher.com/antibody/product/BATF-Antibody-clone-MBM7C7-Monoclonal/46-9860-42
PE-Cyanine7–anti-T-bet: www.thermofisher.com/antibody/product/T-bet-Antibody-clone-eBio4B10-4B10-Monoclonal/25-5825-82
PE–anti-TOX: www.thermofisher.com/antibody/product/TOX-Antibody-clone-TXRX10-Monoclonal/12-6502-82
Alexa Fluor 647–goat anti-rabbit IgG (H+L): www.thermofisher.com/antibody/product/Goat-anti-Rabbit-IgG-H-L-Highly-Cross-Adsorbed-Secondary-Antibody-Polyclonal/A-21245
Alexa Fluor Plus 405–goat anti-rabbit IgG (H+L): www.thermofisher.com/antibody/product/Goat-anti-Rabbit-IgG-H-L-Highly-Cross-Adsorbed-Secondary-Antibody-Polyclonal/A48254
Brilliant Violet 605–anti-Ly108: www.bdbiosciences.com/en-au/products/reagents/flow-cytometry-reagents/research-reagents/single-color-antibodies-ruo/bv605-mouse-anti-mouse-ly-108.745250
Alexa Fluor 647–anti-active caspase-3: www.bdbiosciences.com/en-au/products/reagents/flow-cytometry-reagents/research-reagents/single-color-antibodies-ruo/alexa-fluor-647-rabbit-anti-active-caspase-3.560626
Alexa Fluor 647–anti-BrdU: www.bdbiosciences.com/en-us/products/reagents/microscopy-imaging-reagents/immunofluorescence-reagents/alexa-fluor-647-mouse-anti-brdu.560209
VioletFluor 450–anti-IFNγ: www.tonbobio.com/products/violetfluor-450-anti-mouse-ifn-gamma-xmg1-2
APC–anti-RUNX3/CBFA3: www.rndsystems.com/products/human-mouse-runx3-cbfa3-apc-conjugated-antibody-527327_ic3765a
Alexa Fluor 647–anti-TCF1: www.cellsignal.com/products/antibody-conjugates/tcf1-tcf7-c63d9-rabbit-mab-alexa-fluor-647-conjugate/6709
APC–anti-pS6 (S235/236): www.cellsignal.com/products/antibody-conjugates/phospho-s6-ribosomal-protein-ser235-236-d57-2-2e-xp-rabbit-mab-apc-conjugate/14733

4. The following antibodies for immunoblot have been validated for the specificity and application by the manufacturers (see detailed reference on the website).
anti-β-Actin: www.sigmaaldrich.com/US/en/product/sigma/a2228
anti-Rbpsuh (also known as RBPJ): www.cellsignal.com/products/primary-antibodies/rbpsuh-d10a4-xp-rabbit-mab/5313?site-search-type=Products&N=4294956287&Ntt=5313s&fromPage=plp&_requestid=5443643
HRP-conjugated anti-mouse IgG: https://www.promega.com/products/protein-detection/primary-and-secondary-antibodies/anti_mouse-igg-h-and-l-hrp-conjugate/?catNum=W4021
HRP-conjugated anti-Rabbit IgG: https://www.promega.com/products/protein-detection/primary-and-secondary-antibodies/anti-rabbit-igg-h-and-l-hrp-conjugate/?catNum=W4011

Eukaryotic cell lines

Policy information about cell lines and Sex and Gender in Research

Cell line source(s)	The Plat-E cell line was provided by Y.-C. Liu (La Jolla Institute of Immunology). The B16-OVA cell line was provided by D. Vignali (University of Pittsburgh). The B16-F10 and E.G7-OVA (derivative of EL4) cell lines were purchased from ATCC. B16-hCD19 cell line was constructed by transducing B16 F10 cells with an amphotropic virus containing human CD19 (hCD19) and sorting cells with top 10% hCD19 expression. The Lewis lung carcinoma (LLC) cell line was purchased from ATCC, and the LLC-OVA cell line was produced by transduction of the parental LLC cell line with the pMIG-II-neo-mOVA containing OVA protein fused with GFP, followed by sorting of GFP-expressing cells.
Authentication	The cell lines used were not authenticated.
Mycoplasma contamination	The cell lines were not tested for mycoplasma contamination.
Commonly misidentified lines (See ICLAC register)	No commonly misidentified cell lines were used.

Animals and other research organisms

Policy information about studies involving animals; ARRIVE guidelines recommended for reporting animal research, and Sex and Gender in Research

Laboratory animals	Mice were housed and bred at the St. Jude Children's Research Hospital Animal Resource Center in specific pathogen-free conditions. Mice were on 12-hour light/dark cycles that coincide with daylight in Memphis, TN, USA. The St. Jude Children's Research Hospital Animal Resource Center housing facility was maintained at 20–25°C and 30–70 % humidity. C57BL/6, OT-I, pmel and Rosa26-Cas9-transgenic mice were purchased from The Jackson Laboratory. Human CD19 CAR-transgenic mice (T cells express CARs that consist of anti-human CD19 (human CD19) scFv fragments, CD8 transmembrane domain and 4-1BB-CD3ζ signaling tail) were provided by T. Geiger. We crossed Rosa26-Cas9 knock-in mice with OT-I, pmel or CAR-transgenic mice to generate OT-I-Cas9, pmel-Cas9 or CAR-transgenic-Cas9 mice that express Cas9 in antigen-specific CD8 T cells. Both male and female mice were used for analysis and quantification. Sex-matched mice were used at 7–16 weeks old unless otherwise noted.

Wild animals	The study did not involve wild animals.
Reporting on sex	Both male and female mice were included in all analyses reported in this manuscript, as there were no differences between sexes observed in any of our biological or functional assays.
Field-collected samples	The study did not involve samples collected from the field.
Ethics oversight	Experiments and procedures were approved by and performed in accordance with the Institutional Animal Care and Use Committee (IACUC) of St. Jude Children's Research Hospital.

Note that full information on the approval of the study protocol must also be provided in the manuscript.

Flow Cytometry

Plots

Confirm that:

☒ The axis labels state the marker and fluorochrome used (e.g. CD4-FITC).

☒ The axis scales are clearly visible. Include numbers along axes only for bottom left plot of group (a 'group' is an analysis of identical markers).

☒ All plots are contour plots with outliers or pseudocolor plots.

☒ A numerical value for number of cells or percentage (with statistics) is provided.

Methodology

Sample preparation	The spleens, peripheral lymph nodes (PLNs) and tumor draining lymph nodes (tdLNs) were gently grinded under nylon mesh using the flat end of a 3-mL syringes. Red blood cells were removed using ACK lysis buffer, followed by washing cells with isolation buffer (HBSS containing 2% FBS). To isolate tumor infiltrating lymphocytes (TILs), B16-OVA melanoma, EG.7-OVA or LLC-OVA tumors were excised, minced, and digested with 0.5 mg/ml collagenase IV (Worthington) plus 200 IU/ml DNase I (Sigma) for 1 h at 37 ºC and then passed through 70-um filters to remove undigested tumor tissue. TILs were then isolated by density-gradient centrifugation over Percoll (Life Technologies).
Instrument	LSRII, Symphony A3 or LSR Fortessa (BD Biosciences); Reflection cell sorter (i-Cyt).
Software	BD FACSDIva software (version 8) was used to collect flow cytometry data on LSRII, Symphony A3 or Fortessa cytometers (BD Biosciences). FlowJo v10 (TreeStar) for FACS results.
Cell population abundance	The purities of the sorted cells were more than 98%.
Gating strategy	For all experiments, FSC-A vs. SSC-A gates was used to identify population targeted viable cells. Singlet cells were separated from doublets using FSC-A vs. FSC-H gating. Live viability dye was used to eliminate dead cells. Target populations were further determined by specific antibodies, which were able to distinguish from negative populations.

☒ Tick this box to confirm that a figure exemplifying the gating strategy is provided in the Supplementary Information.
