# Supplementary material for: Single-cell CRISPR screens in vivo map T cell fate regulomes in cancer
Source: Nature. 2023 Nov 15;624(7990):154–63. doi: 10.1038/s41586-023-06733-x (PMC10700132; doi:10.1038/s41586-023-06733-x)
Supplement: Supplementary file 3 — This zipped file contains Supplementary Tables 1–13 and a Supplementary Table guide. [file 41586_2023_6733_MOESM3_ESM.zip › Supplementary Table Guide.pdf]

Supplementary Table 1. Gene list in the scCRISPR library and the re-analysis results of RNA-seq and ATAC-seq public datasets. This file contains the comparisons that were performed between Tpex vs. Tex (Chen et al., 2019; Miller et al., 2019; Yao et al., 2021) or early exhaustion vs. late exhaustion cells (Philip et al., 2017). In Chen et al., 2019 dataset, CAR T cells (CAR T) and endogenous $CD8^+$ T cells (Endo) were both analyzed. UP or DN indicates that gene expression, open chromatin region (OCR) accessibility or motif enrichment was significantly higher or lower in Tpex (or early exhaustion), respectively. UP_DN in differential accessibility (DA) columns indicates that higher and lower accessible OCRs of the gene were both detected. Blank spaces indicate that no significant upregulation or downregulation was observed. DE, differential expression; ME, motif enrichment; Exh, exhaustion.

Supplementary Table 2. Gene list and sgRNA sequences of the scCRISPR library. This file contains all the sgRNAs in the library, listed in two columns (gseq_1 and gseq_2). The library consists of 720 sgRNAs targeting 180 transcription factors (4 sgRNAs per gene), as well as 80 non-targeting control sgRNAs.

Supplementary Table 3. Genes in each co-functional module. This file contains the classifications of 172 perturbations that had ≥48 cells into nine co-functional modules.

Supplementary Table 4. Genes in each co-regulated gene program. This file contains the classifications of target genes from 172 perturbations into four co-regulated gene programs.

Supplementary Table 5. The regulatory effect of each transcription factor (TF) on other TFs in M2, M3, M5, M6, M7, and M8. This file contains the log_2FC (perturbation vs. sgNTC) of each perturbed TF on the other TF genes in co-functional M2, M3, M5, M6, M7, and M8. M, module.

Supplementary Table 6. Tpex and Tex cell state-specific regulators identified in scCRISPR screening. This file contains the log_2FC of the sgRNAs of each transcription factor in the comparisons of each state (in Tpex1, Tpex2, Tex1 and Tex2) versus all the other three states.

Supplementary Table 7. The sgRNA deletion efficiency of *in vivo* OT-I cells. This file contains the indel (insertion and deletion) mutation rate of each sgRNA from intratumoral OT-I cells.

Supplementary Table 8. Positive and negative regulators between Tpex and Tex cell states identified in scCRISPR screening. This file contains the log_2FC of the sgRNAs of each

transcription factor in the comparisons between Tpex and Tex states (Tpex1 versus Tpex2, Tex1 versus Tpex2 and Tex1 versus Tex2).

Supplementary Table 9. *In vivo* genetic interaction CRISPR screening results in sgNTC and sg*Ikzf1* OT-I cells. This file contains two comparisons (intratumoral Tpex versus intratumoral Tex and intratumoral Tpex versus input) in sgNTC (indicated as WT) and sg*Ikzf1* (indicated as KO) OT-I cell screens.

Supplementary Table 10. *In vivo* genetic interaction CRISPR screening results in sgNTC and sg*Ets1* OT-I cells. This file contains two comparisons (intratumoral Tex versus intratumoral Tpex and intratumoral tumor Tex versus input) in sgNTC (indicated as WT) and sg*Ets1* (indicated as KO) OT-I cell screens.

Supplementary Table 11. Gene set enrichment analysis of transcriptome data of sg*Rbpj* vs. sgNTC OT-I cells. This file contains top enriched pathways of sg*Rbpj* versus sgNTC OT-I cells in gene set enrichment analysis (GSEA) using Hallmark (profiled by scCRISPR screening) and C7 immunological gene sets (profiled by the sg*Rbpj* versus sgNTC scRNA-seq experiment). The gene signatures are ranked by normalized enrichment score (NES). ES, enrichment score; NOM, normalized; FDR, false discovery rate.

Supplementary Table 12. *In vivo* genetic interaction CRISPR screening results in sgNTC and sg*Rbpj* OT-I cells. This file contains three comparisons (tumor versus spleen, intratumoral Tex versus input and intratumoral Tex versus intratumoral Tpex) in sgNTC (indicated as WT) and sg*Rbpj* (indicated as KO) OT-I cell screens.

Supplementary Table 13. sgRNA sequences used in this study. This file contains the sgRNA sequences used in Figs. 2–5.
